# CKIP-1 limits foam cell formation and inhibits atherosclerosis by promoting degradation of Oct-1 by REGγ

**DOI:** 10.1038/s41467-018-07895-3

**Published:** 2019-01-25

**Authors:** Jiao Fan, Lifeng Liu, Qingyan Liu, Yu Cui, Binwei Yao, Minghua Zhang, Yabing Gao, Yesheng Fu, Hongmiao Dai, Jingkun Pan, Ya Qiu, Cui Hua Liu, Fuchu He, Yu Wang, Lingqiang Zhang

**Affiliations:** 1 State Key Laboratory of Proteomics, National Center of Protein Sciences (Beijing), Beijing Institute of Lifeomics, Beijing, 100850 China; 2 Institute of Geriatrics, National Clinical Research Center of Geriatrics Disease, Chinese PLA General Hospital, Beijing, 100853 China; 3 Department of Cardiology, Chinese PLA General Hospital, Beijing, 100853 China; 4 Center of Therapeutic Research for Liver Cancer, 302 Military Hospital of China, Beijing, 100039 China; 5 Department of Experimental Pathology, Beijing Institute of Radiation Medicine, Beijing, 100850 China; 6 Clinical Pharmacy Laboratory, Chinese PLA General Hospital, Beijing, 100853 China; 7 CAS Key Laboratory of Pathogenic Microbiology and Immunology, Institute of Microbiology, Chinese Academy of Sciences, Beijing, 100101 China

**Keywords:** Foam cells, Atherosclerosis

## Abstract

Atherosclerosis-related cardiovascular diseases are the leading cause of mortality worldwide. Macrophages uptake modified lipoproteins and transform into foam cells, triggering an inflammatory response and thereby promoting plaque formation. Here we show that casein kinase 2-interacting protein-1 (CKIP-1) is a suppressor of foam cell formation and atherosclerosis. *Ckip-1* deficiency in mice leads to increased lipoprotein uptake and foam cell formation, indicating a protective role of CKIP-1 in this process. Ablation of *Ckip-1* specifically upregulates the transcription of scavenger receptor LOX-1, but not that of CD36 and SR-A. Mechanistically, CKIP-1 interacts with the proteasome activator REGγ and targets the transcriptional factor Oct-1 for degradation, thereby suppressing the transcription of LOX-1 by Oct-1. Moreover, *Ckip-1*-deficient mice undergo accelerated atherosclerosis, and bone marrow transplantation reveals that *Ckip-1* deficiency in hematopoietic cells is sufficient to increase atherosclerotic plaque formation. Therefore, CKIP-1 plays an essential anti-atherosclerotic role through regulation of foam cell formation and cholesterol metabolism.

## Abstract (web summary)

In atherosclerotic plaques, transformation of macrophages into foam cells is a key step in initiating the inflammatory response. Here Fan et al. show that casein kinase 2-interacting protein-1 (CKIP-1) limits foam cell formation and atherosclerosis by preventing expression of the scavenger receptor LOX-1 through REGγ-mediated degradation of Oct-1.

## Introduction

Atherosclerosis is the underlying pathological process of coronary artery disease (CAD) and cerebrovascular disease, which are severe vascular diseases. Atherosclerosis is recognized as a chronic inflammatory disease of large and medium arteries including lipid metabolism disorder and recruitment of immune cells to the artery wall^1^. The crucial early step is the subendothelial retention of lipoproteins that leads to the recruitment of monocytes, which then differentiate into macrophages^2^. Mediated by scavenger receptors, mainly including CD36, scavenger receptor-A (SR-A) or lectin-like oxLDL receptor 1 (LOX-1), macrophages uptake modified lipoproteins such as oxidized LDL (oxLDL) and transform into cholesterol-laden foam cells, triggering a series of inflammatory responses and thereby promoting plaque formation^3^. The regulatory mechanism of this lipoprotein uptake-mediated foam cell formation process remains incompletely understood.

The PH (pleckstrin homology) domain-containing protein CKIP-1 (also known as PLEKHO1) was originally identified as an interacting protein of CK2 kinase and was further shown to play a crucial role in the regulation of tumorigenesis, cell apoptosis, cell morphology, and the actin cytoskeleton^4–8^. In particular, our previous studies showed that CKIP-1 depletion in mice manifests an age-dependent accumulation in bone mass due to increased osteoblast differentiation^9^ and those mice are also susceptible to pressure overload-induced cardiac hypertrophy^10^. Interestingly, CKIP-1 inhibits macrophage proliferation specifically at the late stage after M-CSF stimulation in cultured cells and *Ckip-1*^−/−^ mice spontaneously develop a macrophage-dominated splenomegaly and myeloproliferation^11^, indicating a role of CKIP-1 in macrophage regulation.

Since macrophage plays a critical role in the development of atherosclerosis^12,13^, we hypothesized that CKIP-1 might participate in the regulation of atherogenesis. We therefore generated double knockout mice lacking *Ckip-1* and *Apoe*. Here, we show that knocking out *Ckip-1* causes a significant increase in aortic root macrophage content, increases vascular inflammation, and enhances oxLDL uptake in macrophages, which culminates in heightened plaque burden in *Apoe*^*−/−*^ mice. Mechanistically, CKIP-1 interacts with the proteasome activator REGγ and targets the transcriptional factor Oct-1 for degradation, thereby suppressing the transcription of scavenger receptor LOX-1. Moreover, bone marrow transplantation reveals that *Ckip-1* deficiency in hematopoietic cells is sufficient to increase atherosclerotic plaque formation. Altogether, these findings provide insights to the role of CKIP-1 in the pathogenesis of atherosclerosis.

## Results

### Deletion of *Ckip-1* promotes foam cell formation

**Fig. 1:**
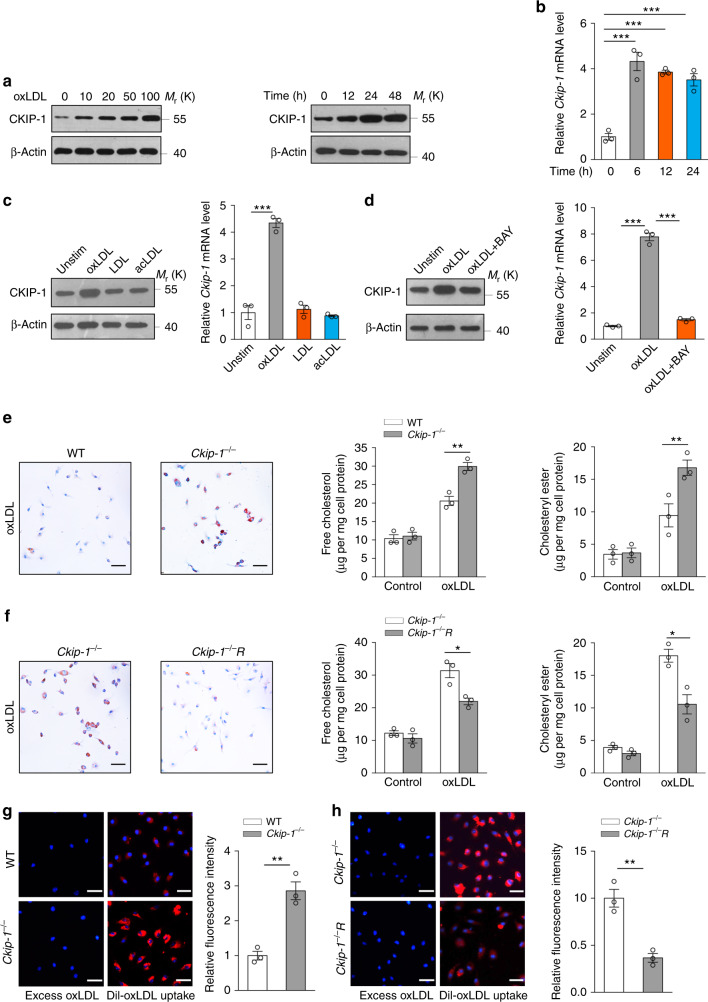
CKIP-1 reduces foam cell formation in macrophages. **a** CKIP-1 expression was assessed by western blot in BMDMs incubated with oxLDL (50 μg per ml) for the indicated time (left) and in BMDMs exposed to different doses of oxLDL for 24 h (right). **b** Real-time PCR analysis of mRNA levels for CKIP-1 in BMDMs after incubation with oxLDL (50 μg per ml) for indicated time. **c** Analysis of CKIP-1 expression in BMDMs treated with oxLDL, LDL, or acLDL (50 μg per ml) for 24 h. **d** BMDMs were treated with or without NF-κB inhibitor BAY11-7082 (10 μM) for 1 h and then stimulated with oxLDL (50 μg per ml) for 24 h. Protein levels and mRNA levels of CKIP-1 were assessed. **e** Increased foam cell formation and accumulation of unesterified cholesterol and cholesteryl ester in *Ckip-1*^*−/−*^ BMDMs after treatment with oxLDL (50 μg per ml) for 24 h. Scale bar, 50 μm. **f** Restoration of CKIP-1 into *Ckip-1*^*−/−*^ BMDMs (*Ckip-1*^−*/−*^R) reduced foam cell formation after treatment with oxLDL (50 μg per ml) for 24 h. Scale bar, 50 μm. **g** Total uptake of Dil-oxLDL was quantified in BMDMs from mice with the indicated genotypes. Scale bar, 25 μm. **h** Restoration of CKIP-1 into *Ckip-1*^*−/−*^ BMDMs reduced induced uptake. Scale bar, 25 μm. Data represent mean ± s.e.m. of *n* = 3 biologically independent experiments (**b**–**h**). *P* values were calculated by one-way ANOVA (**b**) and two-tailed Student’s *t*-test (**c**–**h**). *P < 0.05, **P < 0.01, ***P < 0.001. The precise *P* value and statistics source data are shown in Supplementary Data 2. Unprocessed original scans of blots are shown in Supplementary Fig. 6

We first assessed the possible involvement of CKIP-1 in foam cell formation and found a dose-dependent and time-dependent increase of CKIP-1 protein level in the oxLDL-treated bone marrow-derived macrophages (BMDMs) (Fig. 1a). Treatment of macrophages with oxLDL also upregulated the level of CKIP-1 mRNA (Fig. 1b). Similar results were obtained in peritoneal macrophages (pMΦ) (Supplementary Fig. 1a, b). We found that only oxLDL, but not unmodified LDL or acetylated LDL (acLDL), upregulated CKIP-1 expression on BMDMs (Fig. 1c). Notably, the upregulation of CKIP-1 protein and mRNA by oxLDL was markedly inhibited by the treatment with NF-κB inhibitor BAY11-7082 (Fig. 1d). To explore the role of CKIP-1 in the foam cell formation, wild-type (WT) and *Ckip-1*^*−/−*^ BMDMs were incubated with oxLDL or serum from atherosclerosis-prone apolipoprotein E-deficient (*Apoe*^*−/−*^) mice, which contained atherogenic lipoprotein to induce foam cell formation. *Ckip-1*^*−/−*^ BMDMs showed an enhanced foam cell formation and accumulated more cholesteryl ester and free cholesterol compared with WT BMDMs (Fig. 1e, Supplementary Fig. 1c). Importantly, reconstitution of *Ckip-1*^*−/−*^ BMDMs with ectopic CKIP-1 reduced foam cell formation and cholesterol accumulation in macrophages (Fig. 1f, Supplementary Fig. 1d). These results strongly indicate that *Ckip-1* deficiency promotes foam cell formation.

To investigate whether increased uptake of modified forms of LDL could account for enhanced foam cell formation in *Ckip-1*^*−/−*^ macrophages, we performed uptake assays with Dil-labeled oxLDL. Immunofluorescence revealed a 2.5-fold increase of uptake in *Ckip-1*^*−/−*^ BMDMs compared with WT BMDMs (Fig. 1g). The enhanced oxLDL uptake by *Ckip-1*^*−/−*^ macrophages was reversed by restoration of ectopic CKIP-1 expression (Fig. 1h), substantiating a role of CKIP-1 in uptake of modified lipoproteins. When examining whether CKIP-1 is involved in cholesterol efflux, ^3^H-labeled cholesterol tracer was used to analyze the efflux to lipid-poor ApoA1 or HDL. Induction of cholesterol efflux or LXR agonists TO-901317 had no significant effect on the expression of CKIP-1 (Supplementary Fig. 1e, f) and cholesterol efflux to lipid-poor ApoA1 or HDL was comparable in WT and *Ckip-1*^*−/−*^ BMDMs (Supplementary Fig. 1g).

### CKIP-1 diminishes the expression of scavenger receptor LOX-1

**Fig. 2:**
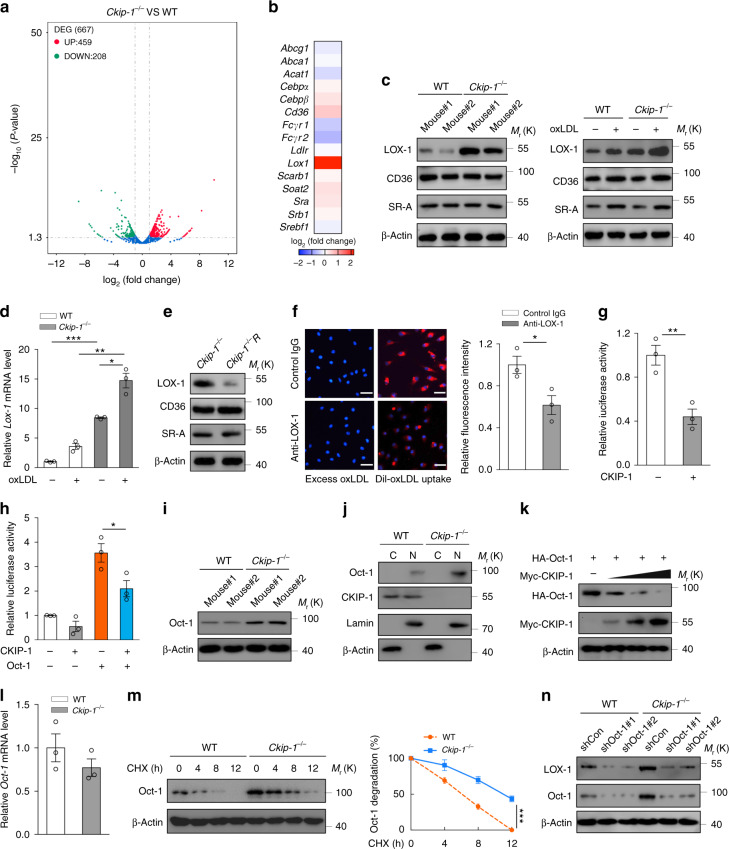
CKIP-1 diminishes Oct-1-mediated LOX-1 expression. **a**, **b** WT and *Ckip-1*^*−/−*^ BMDMs were analyzed RNA sequencing. Identification of the DEGs was graphed in volcano plot (**a**) and selected genes involved in foam cell formation (**b**) are shown as a heatmap. **c** Expression of LOX-1, CD36, and SR-A in WT and *Ckip-1*^*−/−*^ BMDMs after 24 h incubation with or without 20 μg per ml oxLDL. **d** mRNA levels of LOX-1 in BMDMs after incubation with or without oxLDL (20 μg per ml) for 24 h. **e** Expression of LOX-1, CD36, and SR-A in *Ckip-1*^*−/−*^ and *Ckip-1*^*−/−*^R BMDMs. **f** *Ckip-1*^*−/−*^ macrophages were incubated with anti-LOX-1 antibody (10 μg per ml) or control goat IgG for 1 h prior to the addition of Dil-oxLDL or excess unlabeled oxLDL. Total uptake of Dil-oxLDL in each group was quantified. Scale bar, 25 μm. **g**, **h** Luciferase assay in HEK293T cells were transfected with the LOX-1/pGL3 luciferase plasmid and other plasmids as indicated. The luciferase activities were represented after normalization against renilla luciferase activity. **i** Expression analysis of Oct-1 in WT and *Ckip-1*^*−/−*^ BMDMs. **j** The nuclear and cytoplasmic protein samples from WT and *Ckip-1*^*−/−*^ BMDMs were subjected to western blot analysis. β-actin and Lamin were examined to indicate the cytoplasmic (C) and nuclear (N) extracts, respectively. **k** The 293T cells were co-transfected with HA-Oct-1 with increasing amount of Myc-CKIP-1 as indicated. The protein levels were determined by western blot. **l** mRNA levels of Oct-1 in WT and *Ckip-1*^*−/−*^ BMDMs. **m** WT and *Ckip-1*^*−/−*^ BMDMs were treated with cycloheximide (CHX, 10 μg per ml) for the indicated times. Western blot showing relative protein expression levels. **n** Knockdown of Oct-1 expression in WT and *Ckip-1*^*−/−*^ macrophages transfected with Oct-1-shRNA #1, #2 by lentivirus and proteins were analyzed by western blot. Data represent mean ± s.e.m. of *n* = 3 biologically independent experiments (**d**, **f**, **g**, **h**, **l**, **m**). *P* values were calculated by two-tailed Student’s *t*-test (**d**, **f**, **g**, **h**, **l**) and two-way ANOVA (**m**). *P < 0.05, **P < 0.01, ***P < 0.001. The precise *P* value and statistics source data are shown in Supplementary Data 2. Unprocessed original scans of blots are shown in Supplementary Fig. 6

To explore the mechanism of increased foam cell formation in *Ckip-1*-deficient macrophages, we performed RNA sequencing (RNA-seq) in WT and *Ckip-1*^*−/−*^ BMDMs. Total 667 differentially expressed genes (DEGs) were identified by RNA-seq including 459 upregulated and 208 downregulated genes in *Ckip-1*^*−/−*^ BMDMs (Fig. 2a, Supplementary Fig. 2a and Supplementary Data 1). Further analysis with Gene Ontology (GO) and KEGG pathway indicated that these DEGs were enriched for KEGG pathways for cell adhesion molecules and GO terms of multiple biological processes, molecular functions, and signaling pathways in *Ckip-1*-deficient macrophages (Supplementary Fig. 2b, c). Deletion of *Ckip-1* did not affect the expression levels of either ATP-binding cassette transporters responsible for cholesterol efflux, including ABCA1, ABCG1, and SR-BI (Supplementary Fig. 3a–c) or the enzyme required for cholesterol esterification (Supplementary Fig. 3d). Remarkably, the expression of scavenger receptor LOX-1 was upregulated by CKIP-1 deficiency (Fig. 2b). There are several major scavenger receptors in macrophages that are critical in active uptake of modified lipoproteins, such as CD36, SR-A, and LOX-1^14^. We found the basal level of LOX-1 was significantly increased in *Ckip-1*^*−/−*^ BMDMs compared with WT BMDMs, while no difference was observed in the expression levels of CD36 and SR-A (Fig. 2c). This phenomenon was confirmed by immunofluorescence measurement (Supplementary Fig. 3e). Under the condition of exposure with oxLDL, deletion of *Ckip-1* further increased LOX-1 expression at both mRNA and protein levels, but exerted no marked effect on CD36 or SR-A expression (Fig. 2c, d and Supplementary Fig. 3f, g). Similar results were obtained in peritoneal macrophages derived from WT and *Ckip-1*^*−/−*^ littermates (Supplementary Fig. 3h). Upon reconstitution of ectopic CKIP-1 in *Ckip-1*^*−/−*^ macrophages, the LOX-1 expression was reduced while the expression of CD36 and SR-A was unaffected by CKIP-1 overexpression (Fig. 2e), demonstrating that CKIP-1 specifically regulates the expression of LOX-1. We used a specific anti-LOX-1 antibody to block the LOX-1-mediated effect. Indeed, uptake of oxLDL was decreased by ~50% in *Ckip-1*^*−/−*^ BMDMs due to the neutralization of LOX-1 (Fig. 2f). Although there are alternative pathways other than scavenger receptor to mediate the uptake of lipoprotein, such as pinocytosis, we observed no difference in pinocytosis between the tested groups (Supplementary Fig. 3i). These data suggest that deletion of *Ckip-1* augments the cellular uptake of oxLDL, at least in part, through upregulation of LOX-1.

Overexpression of CKIP-1 significantly inhibited the activity of a luciferase reporter driven by LOX-1 promoter region (Fig. 2g), suggesting that CKIP-1 inhibits the transcription of LOX-1 directly. We next investigated the mechanism of how CKIP-1 regulates the transcription of LOX-1. NF-κB has been reported to play a critical role in inflammation and transcriptionally regulate LOX-1; however, CKIP-1 did not affect the expression of NF-κB in macrophages (Supplementary Fig. 3j) and NF-κB inhibitor BAY 11-7082 exerted no marked effect on the upregulated expression of LOX-1 in *Ckip-1*^*−/−*^ BMDMs (Supplementary Fig. 3k). There is evidence for octamer-binding transcription factor 1 (Oct-1, encoded by *POU2F1*) to foster LOX-1 expression^15^. We observed that the overexpression of Oct-1 significantly enhanced the reporter activity of LOX-1 promoter and co-expression of CKIP-1 repressed the transcriptional factor activity of Oct-1 towards LOX-1 promoter (Fig. 2h). Interestingly, the expression of Oct-1 protein was increased in *Ckip-1*^*−/−*^ BMDMs compared with WT cells (Fig. 2i), suggesting that CKIP-1 regulates Oct-1 expression level rather than NF-κB level. Cell fractionation analysis showed that endogenous Oct-1 was mainly localized in the nucleus of BMDMs and deletion of *Ckip-1* resulted in an increase of nuclear Oct-1 (Fig. 2j). Ectopic expression of CKIP-1 resulted in a marked reduction of Oct-1 protein in a dose-dependent manner (Fig. 2k). Importantly, the levels of Oct-1 mRNA were comparable in WT and *Ckip-1*^*−/−*^ BMDMs (Fig. 2l). Protein half-life analysis showed that Oct-1 protein was more stable in *Ckip-1*^*−/−*^ BMDMs as compared with WT BMDMs (Fig. 2m), suggesting that CKIP-1 negatively regulates the stability of Oct-1 protein. Knockdown of Oct-1 by two independent shRNAs in *Ckip-1*^*−/−*^ macrophages reduced the expression of LOX-1 (Fig. 2n), substantiating a role of Oct-1 in CKIP-1-mediated repressive effect on LOX-1 expression.

### CKIP-1 promotes proteasomal degradation of Oct-1 via REGγ

**Fig. 3:**
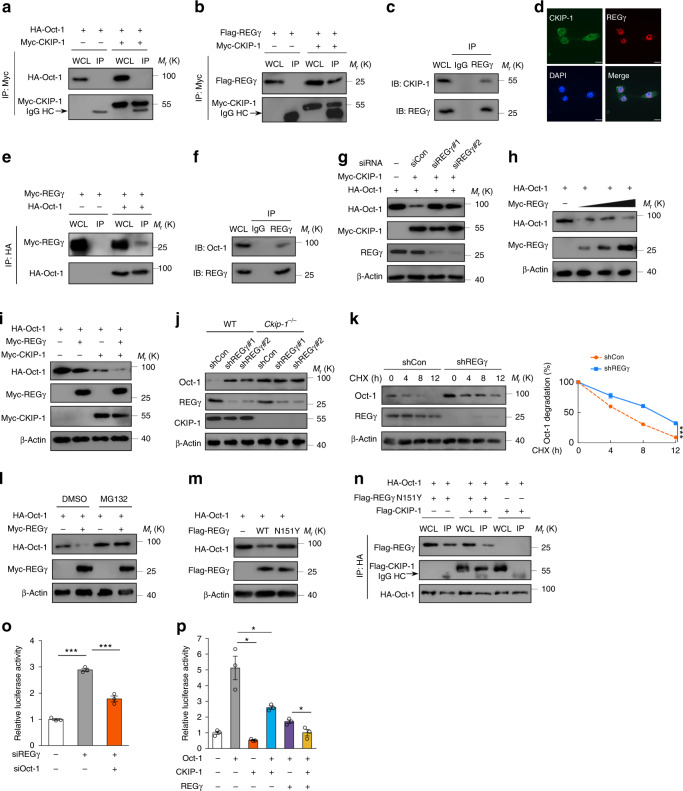
CKIP-1 regulates the stability of Oct-1 via REGγ-dependent proteasome degradation. **a** Co-IP of Oct-1 and CKIP-1. HA-Oct-1 and Myc-CKIP-1 were transfected into HEK293T cells. IP immunoprecipitation, WCL whole cell lysate. **b** Co-IP of CKIP-1 and REGγ. Flag-REGγ and Myc-CKIP-1 were transfected into HEK293T cells. **c** Co-IP interaction assay for the endogenous interaction between CKIP-1 and REGγ in BMDMs. **d** Immunofluorescence assays show the co-localization of CKIP-1 and REGγ in BMDMs. Scale bar, 25 μm. **e** Co-IP of Oct-1 and REGγ. Myc-REGγ and HA-Oct-1 were transfected into HEK293T cells. **f** Co-IP interaction assay for the endogenous interaction between REGγ and Oct-1 in BMDMs. **g** HA-Oct-1, Myc-CKIP-1, and REGγ siRNA were cotransfected into HeLa cells and proteins were analyzed by western blot. **h** HEK293T cells were co-transfected with HA-Oct-1 with increasing amount of Myc-REGγ as indicated. **i** HEK293T cells were transfected with HA-Oct-1, Myc-REGγ, Myc-CKIP-1 or together as indicated. **j** Knockdown of REGγ levels in WT and *Ckip-1*^*−/−*^ macrophages transfected with REGγ-shRNA #1, #2 by lentivirus. **k** Knockdown of REGγ in WT macrophages followed by CHX (10 μg per ml) treatment for the indicated times. **l** The transfected HEK293T cells were treated with MG132 (20 μM) as indicated for 8 h before harvest, and cell lysates were subjected to western blot. **m** HEK293T cells were transfected with HA-Oct-1 and WT REGγ or N151Y mutant, and cell lysates were subjected to western blot. **n** HEK293T cells were transfected with HA-Oct-1, Flag-CKIP-1, and Flag-REGγ N151Y mutant as indicated, and Co-IP assays were performed. **o** HeLa cells were transfected with siRNA against REGγ or Oct-1 as indicated and then cells were transfected with Renilla luciferase plasmids together with LOX-1/pGL3 luciferase plasmid. **p** Luciferase assay in HEK293T cells were transfected with the LOX-1/pGL3 luciferase plasmid and CKIP-1, Oct-1, REGγ plasmids as indicated. Data represent mean ± s.e.m. of *n* = 3 biologically independent experiments (**k**, **o**, **p**). *P* values were calculated by two-way ANOVA (**k**) and two-tailed Student’s *t*-test (**o**, **p**). *P < 0.05, **P < 0.01, ***P < 0.001. The precise *P* value and statistics source data are shown in Supplementary Data 2. Unprocessed original scans of blots are shown in Supplementary Fig. 6

We therefore explored the mechanism of the destabilization of Oct-1 protein by CKIP-1. Initially, we performed a co-immunoprecipitation (Co-IP) assay to test whether CKIP-1 interacts with Oct-1 directly, but failed to detect an obvious binding (Fig. 3a). To identify the possible mediator linking CKIP-1 and Oct-1, we performed a yeast two-hybrid assay with CKIP-1 as the bait to screen a human adult brain library. One of the positive clones encoded the full-length REGγ (regulator γ of proteasome, also known as PA28γ, PSME3) (Supplementary Table 1), a member of 11S family of proteasome activator of the core proteasome^16^. Recent studies indicate that REGγ can target intact proteins for degradation in ubiquitin- and ATP-independent manner^17^. Several transcriptional factors or coactivators, such as SRC-3, p53, and c-Myc, have been identified as REGγ targets^18–20^. We then examined the interaction between CKIP-1 and REGγ, and Co-IP assays readily revealed an association between CKIP-1 and REGγ (Fig. 3b). The endogenous interaction between CKIP-1 and REGγ was also observed in BMDMs (Fig. 3c). Immunofluorescence analysis showed that endogenous CKIP-1 was expressed and localized in both the nucleus and the cytoplasm, whereas REGγ was found mainly in the nucleus and co-localized with CKIP-1 (Fig. 3d). Strikingly, we found that REGγ interacted with Oct-1 as well (Fig. 3e, f). We then asked whether REGγ contributes to the CKIP-1-mediated regulation of Oct-1 protein stability, and expectedly, we found that knockdown of REGγ abrogated the CKIP-1-mediated downregulation of Oct-1 protein levels (Fig. 3g). As a support, the overexpression of REGγ promoted the degradation of Oct-1, and CKIP-1 and REGγ had the synergic function on Oct-1 regulation (Fig. 3h, i). Furthermore, knockdown of REGγ by shRNA caused an increase in Oct-1 protein levels in macrophages (Fig. 3j). In addition, knockdown of REGγ prolonged the half-life of Oct-1 protein in macrophages (Fig. 3k). Treatment with MG132, a potent proteasome inhibitor, completely blocked the REGγ-mediated Oct-1 degradation (Fig. 3l), indicating that REGγ-promoted Oct-1 degradation is dependent on the proteasome activity. Ectopic expression of WT REGγ but not its inactive mutant REGγ-N151Y promoted the degradation of Oct-1 (Fig. 3m). The interaction between CKIP-1 and Oct-1 could not be detectable until REGγ was introduced into the cells (Fig. 3n). In the presence of REGγ, both CKIP-1 and Oct-1 could be co-immunoprecipitated with REGγ (Fig. 3n). Taken together, these findings indicate that the CKIP-1-dependent destabilization of Oct-1 protein is mediated, at least partially, by the interaction of CKIP-1 with the proteasome activator REGγ.

Because REGγ can promote the degradation of Oct-1, we examined whether REGγ affects Oct-1-mediated expression of LOX-1 using a luciferase reporter gene assay. We found that knockdown of REGγ significantly increased the activity of LOX-1 promoter, which could be reversed by Oct-1 knockdown (Fig. 3o). Moreover, REGγ could suppress the Oct-1-mediated transcriptional response of LOX-1 promoter and the repressive effect could be further enhanced by CKIP-1 (Fig. 3p). These results demonstrate that CKIP-1 cooperates with REGγ to repress the Oct-1-mediated transcription of LOX-1. The role of Oct-1 in transcriptional regulation has been described for a number of target genes^21–23^. We also detected the regulation of CKIP-1 and REGγ on other Oct-1 targets, such as Cdx-2, interleukin-2 (IL-2), and HMGB3. Deletion of CKIP-1 or knockdown of REGγ in macrophages upregulated the expression of HMGB3, but exerted no marked effect on the expression of Cdx-2 or IL-2 (Supplementary Fig. 3l, m), which may be cell type-specific or tissue-specific genes regulated by Oct-1.

Our previous studies demonstrated that *Ckip-1*^*−/−*^ mice spontaneously developed splenomegaly with enlarged lymphoid follicles, and flow cytometry data revealed that the number of splenic macrophages and monocytes, but not T or B cells, were significantly increased in *Ckip-1*^*−/−*^ mice compared to WT littermates^11^. We also performed complete blood cell analysis of peripheral blood from WT and *Ckip-1*^*−/−*^ mice and observed no difference in red blood cells, total white blood cells, or neutrophils levels between the tested groups (Supplementary Fig. 3n).

### Loss of *Ckip-1* promotes atherosclerosis

**Fig. 4:**
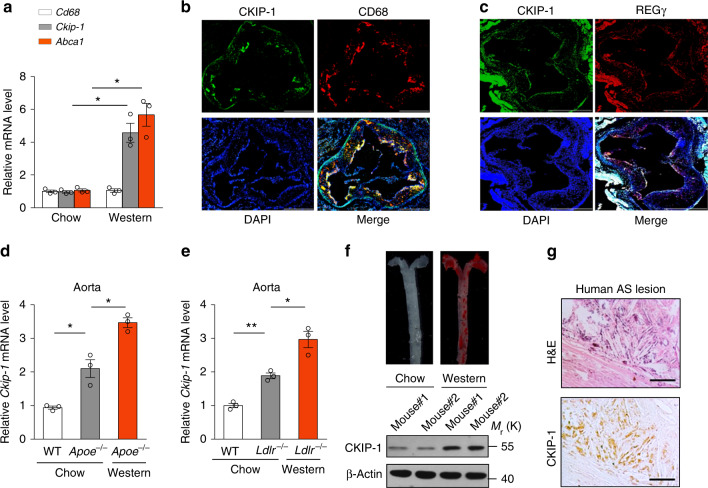
CKIP-1 is highly expressed in atherosclerotic lesion. **a** mRNA levels in pMΦ for CD68, CKIP-1, and ABCA1 (control gene) were compared between chow-fed and Western diet-fed *Apoe*^*−/−*^ mice. **b** CKIP-1 expression was detected by immunofluorescence staining and located with CD68 in *Apoe*^*−/−*^ mouse atherosclerotic lesions (CKIP-1, green; CD68, red; DAPI, blue). Scale bar, 500 μm. **c** Immunofluorescence staining for CKIP-1 and REGγ in *Apoe*^*−/−*^ mouse atherosclerotic lesions (CKIP-1, green; REGγ, red; DAPI, blue). Scale bar, 400 μm. **d** CKIP-1 mRNA levels were compared between the aortic arches of chow-fed WT mice and *Apoe*^*−/−*^ mice, and between the aortic arches of *Apoe*^*−/−*^ mice fed chow or a Western diet. **e** CKIP-1 mRNA levels were compared between the aortic arches of chow-fed WT mice and *Ldlr*^*−/−*^ mice, and between the aortic arches of *Ldlr*^*−/−*^ mice fed chow or a Western diet. **f** Atherosclerotic lesions of chow-fed and western-diet-fed *Apoe*^*−/*−^ mice were stained by Oil Red O. CKIP-1 expression in aortic arch of each group was detected by western blot. **g** Immunohistochemical staining for CKIP-1 in human autopsy atherosclerotic plaques, also stained with H&E to show histological features of the plaque. Scale bar, 50 μm. Data represent mean ± s.e.m. of *n* = 3 biologically independent experiments (**a**, **d**, **e**). *P* values were calculated by two-tailed Student’s *t*-test (**a**, **d**, **e**). *P < 0.05, **P < 0.01, ***P < 0.001. The precise *P* value and statistics source data are in Supplementary Data 2. Unprocessed original scans of blots are shown in Supplementary Fig. 6

Based on the above findings, we hypothesized that deletion of *Ckip-1* might promote atherosclerosis. Thioglycollate-elicited pMΦ isolated from Western diet-fed *Apoe*^*−/−*^ mice, which is commonly used for foam cells formation in vivo, showed much higher expression of CKIP-1 mRNA compared with macrophages from chow-fed mice (Fig. 4a). We found that CKIP-1 was expressed in the CD68-positive macrophages in aortic sinus plaques of *Apoe*^*−/−*^ mice (Fig. 4b). The co-localization of CKIP-1 and REGγ was also observed in the mouse atherosclerotic lesions (Fig. 4c). Compared with the WT mice, the expression of CKIP-1 mRNA in the aortic arch, which is the second atherosclerosis-prone site in mice, was more abundant in *Apoe*^*−/−*^ mice and was further upregulated in the Western diet-fed mice (Fig. 4d). Similar results were obtained with the *Ldlr*^*−/−*^ mouse model of atherosclerosis (Fig. 4e). Western blot analysis also revealed that CKIP-1 was highly expressed in Western diet-fed *Apoe*^*−/−*^ mice compared with chow-fed mice (Fig. 4f). Importantly, CKIP-1 was also expressed in human atherosclerotic lesions (Fig. 4g), suggesting that CKIP-1 expression is a common feature of mouse and human atherosclerotic plaques.

**Fig. 5:**
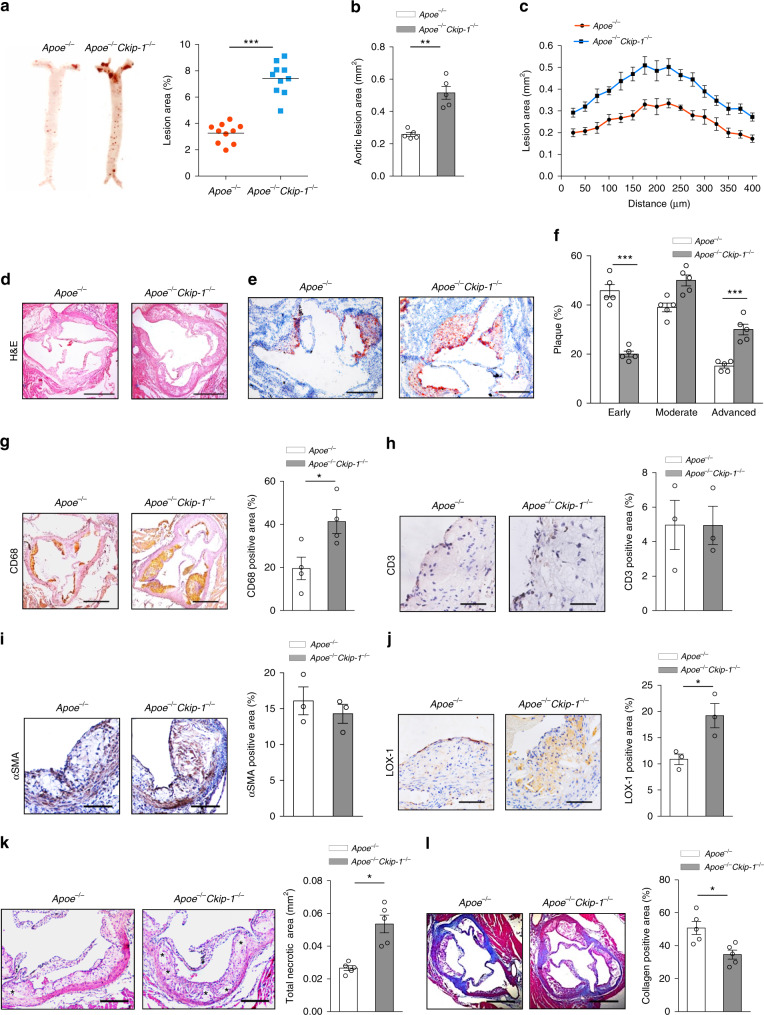
Deficiency of *Ckip-1* leads to severe atherosclerosis in *Apoe*^*−/−*^ mice. **a** *Apoe*^*−/−*^ and *Apoe*^*−/−*^ *Ckip-1*^*−/−*^ littermates were fed a Western diet for 8 weeks. Representative images of en face Oil Red O-stained aortas from mice with the indicated genotypes. *n* = 10. **b** Quantification of lesion area of aortic plaques from each genotype. *n* = 5. **c** Lesion area of atherosclerotic plaques of the aortic roots of *Apoe*^*−/−*^ and *Apoe*^*−/−*^ *Ckip-1*^−*/−*^ mice, presented for each genotype across the 400 μm of the aortic root. *n* = 4. **d** Representative images of cross-sections of the aortic roots from mice with the indicated genotypes. Scale bar, 400 μm. **e** Representative images of cross-sections of the aortic sinus stained with oil red O. Scale bars, 400 μm. **f** The distribution of early, moderate, and advanced plaques based on histological staging of the atherosclerotic lesions. **g** CD68-positive macrophages in lesions from mice with indicated genotypes on a Western diet for 8 weeks. Scale bar, 500 μm. *n* = 3. **h**, **i** Analysis of plaque composition: sections from aortic sinuses were stained with antibodies against CD3 (**h**, T cells; scale bar, 100 μm) or αSMA (**i**, smooth muscle cells; scale bar, 200 μm). *n* = 3. **j** Immunohistochemical detections of LOX-1 in aortas. Scale bars, 100 μm. *n* *=* 3. **k** Representative sections of H&E-stained aortic roots from each group (asterisk indicates necrotic area). Scale bar, 200 µm. The bar graph shows quantification of necrotic areas, *n* = 5. **l** Representative pictures showing the collagen (blue) content from each group. Collagen content statistics are also shown, *n* = 5. Scale bar, 400 µm. Data represent mean ± s.e.m. *P* values were calculated by two-tailed Student’s *t*-test (**a**–**c**, **f**–**l**). *P < 0.05, **P < 0.01, ***P < 0.001. The precise *P* value and statistics source data are in Supplementary Data 2

To explore the possible role of CKIP-1 in atherosclerosis in vivo, *Ckip-1*^*−/−*^ mice were crossed with atherosclerosis-prone *Apoe*^*−/−*^ mice, both of which were in the C57BL/6 backgrounds. Then age- and sex-matched *Apoe*^*−/−*^ *Ckip-1*^−*/−*^ and *Apoe*^*−/−*^ littermates were fed a Western diet for 8 weeks (Supplementary Fig. 4a). Body weights and plasma cholesterol levels of mice with the indicated genotypes before and after being fed a Western diet for 8 weeks were comparable (Supplementary Fig. 4b). The levels of fasting triglycerides and lipoprotein profiles were also not significantly different between both genotypes fed a Western diet (Supplementary Fig. 4c). Despite similar cholesterol profiles, en face analysis of Oil Red O-stained atherosclerotic lesion area revealed an about 2.2-fold increase in *Apoe*^*−/−*^ *Ckip-1*^*−/−*^ mice when compared to *Apoe*^*−/−*^ mice (Fig. 5a). Quantification of lesion burden by cross-sectional analysis of the aorta revealed that loss of CKIP-1 increased the lesion areas (Fig. 5b–e). The lesions were grouped into three categories as previously described^24^ and our analysis showed that *Apoe*^*−/−*^ *Ckip-1*^*−/−*^ plaques had undergone more severe plaque progression (Fig. 5f), indicating that loss of CKIP-1 promotes the progression of atherosclerotic lesions to more advanced stages.

We then conducted a more detailed analysis of aortic root plaque composition. Staining for biomarkers of macrophages (CD68), smooth muscle cells (α-smooth muscle actin, αSMA), or T cells (CD3) confirmed more macrophages in the plaques of *Apoe*^*−/−*^ *Ckip-1*^*−/−*^ mice (Fig. 5g) and no difference in CD3-positive and αSMA-positive areas (Fig. 5h, i). To explore the role of CKIP-1 in cell apoptosis, we stained serial sections from the proximal aorta with terminal deoxynucleotidyl transferase-mediated dUTP nick-end-labeling (TUNEL). The percentage of TUNEL-positive (TUNEL^+^) cells in atherosclerotic lesions showed no difference between the two groups of mice (Supplementary Fig. 4d). The expression of scavenger receptor LOX-1 was upregulated in peritoneal macrophages from Western diet-fed *Apoe*^*−/−*^ *Ckip-1*^*−/−*^ mice (Supplementary Fig. 4e). Furthermore, the expression of LOX-1 in atherosclerotic lesions was significantly increased in the absence of CKIP-1 with no significant difference in expression of CD36 and SR-A (Fig. 5j and Supplementary Fig. 4f, g). Analysis of plaque morphology showed that *Ckip-1* deficiency significantly increased the necrotic core areas (Fig. 5k) and promoted the degradation of collagen (Fig. 5l), which are important features of vulnerable plaques.

### *Ckip-1* deletion increases systemic inflammation and MMP activity

**Fig. 6:**
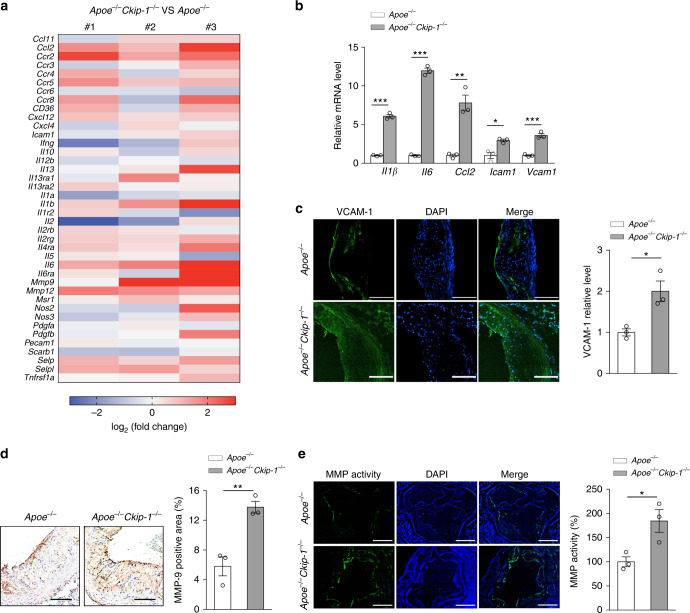
*Ckip-1* deficiency enhances systemic inflammation and matrix metalloproteinase activity. **a** Expression levels of the indicated genes were assessed by using three independent Capital Bio mRNA Array. The fold-change for each gene of *Apoe*^*−/−*^ *Ckip-1*^*−/−*^ with respect to *Apoe*^*−/−*^ mice on a 12-week Western diet was calculated. **b** Relative expression levels of cytokine and chemokine mRNA in lesions from *Apoe*^*−/*−^ mice and *Apoe*^*−/−*^ *Ckip-1*^*−/−*^ mice on a 12-week Western diet were measured by RT-PCR. *n* = 3. **c** Expression of VCAM-1 in lesions from aortic roots. Scale bars, 100 μm. *n* = 3. **d** Immunohistochemical detections of MMP-9 in aortas. Scale bars, 200 µm. *n* = 3. **e** MMP activity was studied by in situ zymography assay in aortas. Scale bars, 400 µm. *n* = 3. Data represent mean ± s.e.m. *P* values were calculated by two-tailed Student’s *t*-test (**b**–**e**). *P < 0.05, **P < 0.01, ***P < 0.001. The precise *P* value and statistics source data are in Supplementary Data 2

We next analyzed the expression of genes involved in vascular inflammation in the aortic wall of *Apoe*^*−/−*^ and *Apoe*^*−/−*^ *Ckip-1*^*−/−*^ mice by mRNA expression array technology. The expression levels of the proinflammatory cytokines including interleukin (IL)-1β, IL-6, and matrix metalloproteinase (MMP)-9 were significantly upregulated in *Apoe*^*−/−*^ *Ckip-1*^*−/−*^ mice (Fig. 6a). Consistent with the array data, statistically significant differences were observed in the mRNA levels of IL-1β, IL-6, and CCL-2 detected by RT-PCR in the absence of CKIP-1 (Fig. 6b). Vascular cell adhesion molecule 1 (VCAM-1) expression by vascular cells is a characteristic feature of atherosclerosis, reflecting the inflammatory state in the plaque^25^. Similar to higher mRNA levels of VCAM-1, increased expression of VCAM-1 positive areas in *Apoe*^*−/−*^ *Ckip-1*^*−/−*^ lesions was observed (Fig. 6b, c). In accordance with the mRNA level (Fig. 6a), there was a significant increase of MMP-9-positive areas and MMP activity assessed by in situ zymography in *Apoe*^*−/−*^ *Ckip-1*^*−/−*^ mice lesions (Fig. 6d, e). As rupture of atherosclerotic plaques has been associated with increased activity of MMPs^26^, enhanced MMP activity may contribute to increased collagen breakdown in lesions of *Apoe*^*−/−*^ *Ckip-1*^*−/−*^ mice.

### CKIP-1 in hematopoietic lineage regulates atherosclerosis

**Fig. 7:**
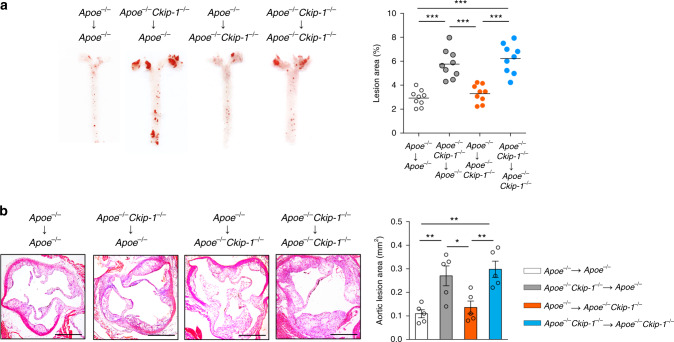
Deletion of *Ckip-1* in hematopoietic cells is responsible for the increased atherosclerosis in *Apoe*^−*/−*^ *Ckip-1*^*−/−*^ mice. **a** Bone marrow transfers were performed in 6-week old lethally irradiated *Apoe*^*−/−*^ and *Apoe*^*−/−*^ *Ckip-1*^*−/−*^ mice followed by reconstitution with bone marrow from *Apoe*^*−/−*^ or *Apoe*^*−/−*^ *Ckip-1*^*−/−*^ mice, respectively. Representative images of Oil Red O-stained aortas of each group chimeras and quantification of lesion area are shown, *n* = 9. **b** Representative images of cross-sections of the aortic roots from each group stained with H&E and quantification of aortic root lesion area are shown. Scale bar, 500 μm. *n* *=* 5. Data represent mean ± s.e.m. *P* values were calculated by two-tailed Student’s *t*-test (**a**, **b**). *P < 0.05, **P < 0.01, ***P < 0.001. The precise *P* value and statistics source data are in Supplementary Data 2

Finally, to determine whether CKIP-1 expression in cells of the hematopoietic lineage or in stromal cells of the arterial compartment regulates atherosclerosis in *Apoe*^*−/−*^ mice, bone marrow transplantation was carried out with lethally irradiated mice as recipients. After 4-week recovery, mice were put on a high-fat diet for 8 weeks (Supplementary Fig. 5a). Successful reconstitution of recipient bone marrow with donor bone marrow was verified by PCR (Supplementary Fig. 5b). Homologous transfers of *Apoe*^−*/−*^ bone marrow into *Apoe*^*−/−*^ mice and of *Apoe*^*−/−*^ *Ckip-1*^*−/−*^ bone marrow into *Apoe*^*−/−*^ *Ckip-1*^*−/−*^ mice were served as controls. The atherosclerotic plaque burden was increased to a similar extent in *Apoe*^*−/−*^ mice receiving *Apoe*^*−/−*^ *Ckip-1*^*−/−*^ bone marrow, whereas no effect on atherosclerosis was observed in chimeras with *Apoe*^*−/−*^ bone marrow in an *Apoe*^*−/−*^ *Ckip-1*^*−/−*^ background (Fig. 7a). In line with these findings was the analysis performed for atherosclerotic lesions at the aortic root (Fig. 7b). There was no significant difference among these groups in body weight, plasma cholesterol, and triglyceride levels (Supplementary Fig. 5c). Collectively, these results of bone marrow chimeras strongly suggested that the hematopoietic expression of CKIP-1 plays a causal role in atherosclerosis.

## Discussion

Lipid metabolism disorder and recruitment of immune cells to the artery wall are the underlying pathological processes of CAD and cerebrovascular disease^1^. Even at very early stages of atherogenesis, many macrophages ingest and process lipoproteins, displaying membrane-bound lipid droplets in the cytoplasm and acquiring a foam cell phenotype. A number of key signaling pathways are highly relevant to foam cell formation, including Ras and MAPK activation, TNF-α and related family members leading to activation of NF-κB and effects of reactive oxygen species (ROS) on signaling^27^.

**Fig. 8:**
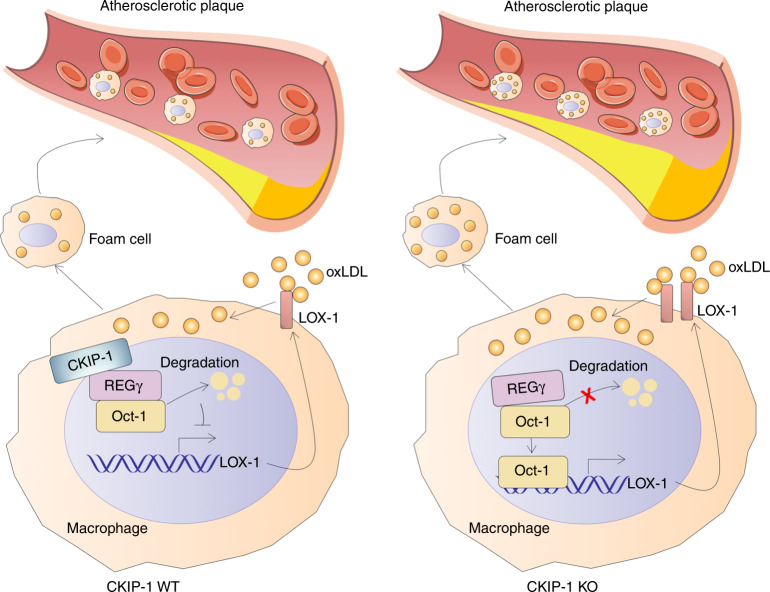
A proposed model for the role of CKIP-1 in atherosclerosis. CKIP-1 regulates the formation of foam cells by coupling proteasome activator REGγ to target the transcription factor Oct-1 for degradation, thereby suppressing the transcription of LOX-1 and the macrophage lipid uptake. On the contrary, *Ckip-1* deficiency results in the increased expression of LOX-1, facilitates uptake of oxLDL and accumulation of cholesterol within the cells, and further results in the promotion of atherosclerosis

CKIP-1 has originally been identified as an interacting protein of CK2 kinase, an ubiquitously expressed member of the PLEKH family which has been implicated in many key cellular processes in diverse cell types. Our previous studies demonstrated that CKIP-1 is a critical regulator of pathological cardiac hypertrophy and macrophage proliferation^11,28^. Here, we establish that CKIP-1 is expressed in mouse and human atherosclerotic plaques and show that genetic deletion of *Ckip-1* promotes atherosclerosis in a hyperlipidemic mouse model. We further show that *Ckip-1* deficiency leads to increased formation of foam cells and inflammation. Aortic plaque burden is significantly higher in *Apoe*^*−/−*^ *Ckip-1*^*−/−*^ mice on Western diet than in *Apoe*^*−/−*^ controls. In addition, we find that *Ckip-1* deletion increases MMPs expression, and may thus support an instable plaque phenotype. Bone marrow transplantation experiments show that hematopoietic cells derived from *Apoe*^*−/−*^ *Ckip-1*^*−/−*^ donors are sufficient to increase atherosclerotic plaque formation when transplanted to recipient mice. In vitro cell assays show that *Ckip-1* deficiency leads to increased intracellular accumulation of CE and to foam cell formation. Mechanistically, CKIP-1 interacts with REGγ and promotes the degradation of Oct-1, thus inhibiting the transcriptional activity of Oct-1 on LOX-1. In this manner, CKIP-1 attenuates cellular oxLDL uptake in macrophages and reduces the formation of foam cells. In contrast, *Ckip-1* deficiency results in the increased expression of LOX-1, facilitates uptake of oxLDL and accumulation of cholesterol within the cells, and further results in the promotion of atherosclerosis. On the basis of these findings, we can assume a protective role for CKIP-1 during foam cell formation and atherosclerosis (Fig. 8).

The current study provides genetic evidence demonstrating that CKIP-1 is not only an inducible protein upon oxLDL (but not unmodified LDL and acLDL) stimulus, but also a brake-like regulator of foam cell formation and atherosclerosis. The underlying mechanism of foam cell formation may consist of an elevated uptake of proatherogenic lipoproteins, or an inability to remove cholesterol from cells resulting from a defective cholesterol efflux^3^. In this regard, we showed that *Ckip-1* deficiency leads to an increased uptake of lipoproteins without obvious effects on cholesterol removal. Early work suggested that uptake of oxidized LDL occurs via scavenger receptors^14^. Strikingly, we identified CKIP-1 as a specific suppressor of the expression of LOX-1, but not that of CD36 and SR-A. LOX-1 was originally identified as a receptor for oxLDL in endothelial cells^29^ and was also expressed in macrophages^30^. Baseline LOX-1 expression is very low in macrophages; however, it can be upregulated under circumstances of pathological stress such as atherosclerosis and plays a critical role in foam cell formation and inflammatory response in atherosclerotic plaques^31^. Accumulating evidence implicate an association between the expression of LOX-1 and the pathophysiology of atherosclerosis. Neutralization of LOX-1 is sufficient to decrease the uptake of oxLDL in *Apoe*^*−/−*^ *Ckip-1*^*−/−*^ macrophages, suggesting that LOX-1 is involved in the inhibition of oxLDL uptake by CKIP-1. Thus, these findings add CKIP-1 into the LOX-1 axis of the complicated network regulating the development of atherosclerosis.

Our results provide insights into the stability control of Oct-1. A recent study showed that the ubiquitin E3 ligase TRIM21 enhances Oct-1 ubiquitylation and proteasomal degradation^32^. Here, we show that CKIP-1-dependent destabilization of Oct-1 protein is mediated, at least partially, by the interaction of CKIP-1 with the proteasome activator REGγ. CKIP-1 coupled the proteasome activator REGγ to directly recruit Oct-1 for proteasomal degradation, which process might be ubiquitin- and ATP-independent since it is the typical working pattern of REGγ. Recent findings revealed crucial roles of REGγ in regulating various processes or diseases, including energy homeostasis, tumorigenesis, inflammatory bowel disorder, rheumatoid arthritis, and host defense^33–37^. The identification of Oct-1 as a substrate of REGγ suggests that REGγ might also play a role in atherosclerosis, which is worthy of further investigations in the future.

In summary, we propose a working model that CKIP-1 couples proteasome activator REGγ to target Oct-1 for degradation, thereby suppressing the transcription of LOX-1. CKIP-1 is an intrinsic negative regulator of macrophage lipid uptake, and thus may act as a brake during foam cell formation and atherosclerosis. These data extended our understandings of CKIP-1 as a regulator of inflammatory response as well as atherogenesis progression, suggesting a potential strategy for atherosclerosis treatment based on targeting Oct-1-LOX-1 axis.

## Methods

### Animal

*Ckip-1*^*−/−*^ mice (C57BL/6 background) were generated and characterized in our laboratory^9^. *Apoe*^*−/−*^ (B6.129P2-*Apoe*^*tm1Unc*^/J) mice (C57BL/6 background) were purchased from the Jackson Laboratory. *Ckip-1*^*−/−*^ mice were intercrossed with *Apoe*^*−/−*^ mice^38^ to generate *Apoe*^*−/−*^ mice and *Apoe*^*−/−*^ *Ckip-1*^*−/−*^ littermate controls. Atherosclerosis was induced by feeding gender-matched 8-week-old *Apoe*^*−/−*^ mice and *Apoe*^*−/−*^ *Ckip-1*^*−/−*^ mice with a Western diet from Harlan Teklad (TD88137) for indicated times. All experimental procedures in mice were approved by the Laboratory Animal Center of Chinese Academy of Military Medical Sciences and complied with all relevant ethical regulations.

### Cells, plasmids, and reagents

HEK293T (ATCC CRL-3216) and HeLa (ATCC CCL-2) were obtained from the American Type Culture Collection (ATCC). Full-length of CKIP-1 and REGγ were constructed by inserting PCR amplified fragments into the related vectors. Detailed construct information is available upon request. Haemagglutinin (HA)-tagged Oct-1 was purchased from Addgene. The protein synthesis inhibitor cycloheximide (CHX) and the NF-κB inhibitor BAY 11-7082 were purchased from Sigma-Aldrich (St. Louis, MO, USA). The proteasome inhibitor MG132 was purchased from Calbiochem (Germany).

### Antibodies

All antibodies were purchased as follows: Anti-CKIP-1 (sc-50225; for immunohistochemical analysis (IHC), 1:100 dilution; for immunofluorescent analysis (IF), 1:1000 dilution; for western blot analysis (WB), 1:500 dilution), anti-Oct-1 (sc-8024; for WB, 1:500 dilution; for immunoprecipitation analysis (IP), 1:50 dilution), anti-ABCG1 (sc-11150; for IF, 1:100 dilution; for WB, 1:200 dilution), anti-Lamin (sc-518013; for WB, 1:200 dilution), anti-SR-B (sc-32342; for IF, 1:100 dilution; for WB, 1:200 dilution), and anti-actin (sc-1616; for WB, 1:1000 dilution) antibodies were purchased from Santa Cruz. Anti-REGγ (ab157157; for WB, 1:500 dilution; for IP, 1:50 dilution; for IF, 1:100 dilution), anti-CD68 (ab125212; for IHC, 1:200 dilution; for IF, 1:200 dilution), anti-CD3 (ab16669; for IHC, 1:100 dilution), anti-SMA (ab9465; for IHC, 1:200 dilution), anti-ABCA1 (ab18180; for IF, 1:200 dilution; for WB, 1:200 dilution), and anti-ACAT-1 (ab168342; for WB, 1:500 dilution) antibodies were purchased from Abcam. Anti-LOX-1 (AF1564; for IHC, 1:200 dilution; for IF, 1:200 dilution; for WB, 1:1000 dilution), anti-CD36 (AF2519; for IHC, 1:200 dilution; for IF, 1:200 dilution; for WB, 1:1000 dilution), anti-SR-A (AF1797; for IHC, 1:200 dilution; for IF, 1:200 dilution; for WB, 1:1000 dilution), anti-MMP-9 (AF909; for IHC, 1:200 dilution), and anti-VCAM-1 (AF2519; for IF, 1:200 dilution) antibodies were purchased from R&D. Anti-HA (M180-3; for WB, 1:1000 dilution) and anti-Myc (M047-3; for WB, 1:1000 dilution) antibodies were purchased from MBL. Anti-Flag (F7425; for WB, 1:1000 dilution) antibody was purchased from Sigma. Anti-NF-κB (8242; for WB, 1:1000 dilution) antibody was purchased from Cell Signaling Technology.

### Bone marrow transplantation

Bone marrow was collected from sex-matched donor mice femur and tibia. Recipient mice were exposed to lethal irradiation with two 5.5 Gy doses (total 11 Gy) at a 4 h interval in order to minimize radiation toxicity and then transplanted with 10^7^ bone marrow cells by tail vein injection. Transplanted mice were then fed with a Western diet for 8 weeks after 4 weeks recovery. Bone marrow reconstitution was confirmed by PCR analysis.

### Atherosclerotic lesion analysis

Mice fed a Western diet for 8 weeks were anesthetized and euthanized. The entire aortas were isolated and were stained with Oil Red O (Sigma-Aldrich, St. Louis, MO, USA) for en face analysis. For the aortic sinus analysis, aortic roots were dehydrated and paraffin embedded and serial cryosections were taken from the region of the proximal aorta through the aortic sinuses and stained with hematoxylin and eosin (H&E). Oil Red O staining for lipids in cryosections of aortic root was performed using Oil Red O staining Kit (Genmed Scientifics Inc., USA) as recommended by manufacturer’s instructions. Morphological analysis of collagen contents in the lesion was stained with Masson’s trichrome. The necrotic core was defined as a clear area that was H&E free. Classification of aortic plaques was carried out according to severity as early stage: lesions with early fatty streaks, moderate stage: moderate lesions with a collagenous cap, and advanced stage: advanced lesions with involvement of the media and increased necrotic area, as described before^24^. Apoptotic cells were labeled by TUNEL using the In Situ Cell Death Detection Kit (Roche, Switzerland) according to manufacturer’s instruction and observed using fluorescence microscopy. MMP activity was studied by in situ zymography assay. Non-fixed aorta sections prepared by cryostat were thawed and incubated using In Situ Zymography Kit (Genmed Scientifics Inc., USA) as recommended by manufacturer’s instructions and observed using fluorescence microscopy.

### Histological analysis of human tissue

Human atherosclerotic and normal aortic tissues were obtained at the time of autopsy from donors, with informed consent and approval from Chinese PLA General Hospital Ethical Committee. Paraffin-embedded aortic tissues were deparaffinized in xylene and re-hydrated following antigen retrieval and washed by phosphate-buffered saline. Endogenous tissue peroxidase activity was quenched by 3% H_2_O_2_, and blocked in bovine serum albumin. The primary antibody for CKIP-1 (Santa Cruz Biotechnology, CA, USA; sc-50225, 1:100 dilution) was incubated overnight. The sections were washed next day and incubated in secondary antibody. The expression of CKIP-1 was visualized by ABC kit (Boster, CA, USA).

### Immunohistochemistry

Cross-sections of the aortic root were stained with primary antibodies followed by HRP-conjugated secondary antibodies and developed with DAB substrate (brown). Images were captured under the Nikon Bx60 microscope connected to a Nikon DP70 camera with Cell-F imaging software (Soft Imaging System) and quantification was performed with Image Pro Plus Software.

### Immunofluorescence

For frozen sections, frozen sections were fixed in acetone, and processed for antibodies according to standard protocols. For cells, cells were fixed in 4% formaldehyde, permeabilized with 0.2% Triton X-100, and blocked with 3% BSA/PBST. Cells were then incubated with primary antibodies. The corresponding secondary antibodies were from CWBIO (Beijing, China). Images were captured and processed using identical settings in the Zeiss LSM 510 Meta inverted Confocal Microscope.

### Lipids analysis and lipoprotein profile measurement

Mice were fasted overnight before blood samples were collected. Plasma was separated by centrifugation and stored at −80 °C. Total and free cholesterol and triglycerides were enzymatically measured with Cholesterol/CE Quantitation Kit II (Biovision, Mountain View, CA, USA) and Triglyceride Quantification Kit (Biovision, Mountain View, CA, USA) as recommended by manufacturer’s instructions. The concentrations of HDL-cholesterol and LDL-cholesterol in plasma were determined using enzymatic colorimetric assays (Zhongsheng Beikong Bio-technology and Science Inc., Beijing, China) according to the manufacturer’s instructions. Cellular protein concentration was assessed using Pierce BCA Protein Assay Kit (Thermo, CA, USA).

### Foam cell formation

Cells were plated on 12-well plates and incubated with oxLDL (Unionbiol, Beijing, China) for 24 h and then the cells were fixed with 4% formaldehyde, stained with oil red O (Sigma-Aldrich, St. Louis, MO, USA) and counterstained with hematoxylin.

### Uptake of oxLDL

BMDMs were incubated with 10 µg per ml fluorescence-labeled oxLDL (Dil-oxDL, Unionbiol, Beijing, China) for 4 h at 37 °C to assess uptake of Dil-oxLDL. We subjected cells to an excess of unlabeled oxLDL (200 µg per ml) as a negative control. Fluorescence intensity was analyzed under a fluorescence microscope and quantified with Image Pro Plus Software.

### Yeast two-hybrid

Yeast two-hybrid screening of CKIP-1 interacting proteins in human adult brain library was performed with the ProQuest^TM^ two-hybrid system (Invitrogen, CA, USA). Briefly, the WW domains plus the HECT domain (aa 236–731) of human Smurf1 were cloned in-frame with the GAL4 DNA binding domain in the vector pDBLeu to create pDBLeu-Smurf1-WH. MaV203 yeast cells were transformed with pDBLeu-Smurf1-WH and human liver cDNA library in pPC86 vector. A total of approximately 1 × 10^6^ independent transformants were analyzed, and clones were selected for positive interactions based on screening for expression of reporter genes His, LacZ, and URA3.

### Pinocytosis and cholesterol efflux assay

Lucifer Yellow CH (Sigma-Aldrich, St. Louis, MO, USA) was dissolved in 10% FBS/RPMI medium at 0.5 mg per ml. The Lucifer Yellow medium was then added to macrophages cultured in 12-well plates. The culture plates were then either maintained on ice, or at 37 °C for 2 h. The wells were drained, washed with ice-cold 0.2%BSA/RPMI medium 3 times and with PBS for 5 times. Triton X-100 (0.05%, 600 μl per well) was added to each well to lyse cells. Fluorescence of the lysate was detected using spectrofluorometer with excitation at 430 nm and emission at 540 nm.

For cholesterol efflux assay, cells were incubated with RPMI media containing in the presence of 2 μCi per ml of ^3^H-cholesterol (Perkin Elmer Life Sciences, Boston, MA) and 50 μg per ml of oxLDL (Unionbiol, Beijing, China) with supplemented 1 μM LXR agonist TO-901317 (Sigma-Aldrich, St. Louis, MO, USA) as required. After equilibration, the cells were incubated with RPMI media containing indicated concentrations of either BSA, or HDL (Unionbiol, Beijing, China), or ApoA1 (Unionbiol, Beijing, China). Radioactivity was quantified in the media and in cells.

### Cell transfections, immunoprecipitation, and immunoblotting

Cells were transfected with various plasmids using TuboFect in vitro transfection reagent (Fermentas, CA, USA) or Lipofectamine 2000 (Invitrogen, CA, USA) reagent according to the manufacturer’s protocol. For immunoprecipitation assays, cells were lysed with HEPES lysis buffer (20 mM HEPES, pH 7.2, 50 mM NaCl, 0.5% Triton X-100, 1 mM NaF, and 1 mM dithiothreitol) supplemented with protease-inhibitor cocktail (Roche, Switzerland). Immunoprecipitations were performed using the indicated primary antibody and protein A/G agarose beads (Santa Cruz Biotechnology, CA, USA) at 4 °C. The immunocomplexes were then washed with HEPES lysis buffer four times. Immunoblot was performed using the standard protocol. Unprocessed original scans of blots are shown in Supplementary Fig. 6.

### Quantitative real-time PCR

Total RNA was isolated using TRIzol reagent (Invitrogen, CA, USA) and reverse-transcribed using ReverTra Ace (Toyobo, Japan). Quantitative real-time PCR was performed using Realtime PCR Master Mix (Toyobo, Japan) and an iQ5 real-time PCR system (Bio-Rad, CA, USA). The data were normalized by β-actin. The primer sequence for RT-PCR used in this study is provided in Supplementary Table 2.

### Small interfering RNA

Small interfering RNA (siRNA) targeted to human REGγ gene was synthesized by Shanghai Gene Pharma Co, Ltd. REGγ siRNA#1: 5′-GAA UCA AUA UGU CAC UCU AUU-3′, siRNA#2: 5′-UCU GAA GGA ACC AAU CUU AUU-3′, Oct-1 siRNA: 5′-CAC CUU ACA CCG AGU AUG U-3′, and negative control siRNA: 5′-UUC UCC GAA CGU GUC ACG U-3′.

### Viral infection

Mouse CKIP-1 cDNAs were inserted into murine stem cell virus (MSCV)-IRESGFP or (MSCV)-IRES-Puro vector for overexpression assay, Oct-1 and REGγ shRNAs were inserted into U6-Puro-GFP vector for knockdown assays. CKIP-1 vectors, Oct-1-lentiviral shRNA#1 (5′-GCT GCT CAG TCT TTA AAT GTA CTC-3′), shRNA#2 (5′-CAG TGA AGA GTC GGG AGA TTC CTC-3′) and REGγ-lentiviral shRNA#1 (5′-GGA GGA AAC AGT TGC TGA ACT-3′), shRNA#2 (5′-GGA AAC AGT TGC TGA ACTA-3′) were transfected with packing plasmids into 293T cells for 2 days, and virus particles were used to infect macrophages as indicated.

### Reporter gene assays

The 3058-bp (−3000 to +58) LOX-1 promoter was amplified from genomic DNA and then cloned into the pGL3 basic vector (Promega, Madison, WI, USA). 293T cells (5 × 10^4^ cells per well in 24-well plates) were transfected with LOX-1 promoter construct plasmids, phRL-TK vector with or without pcDNA3.1-CKIP-1 or pCGN-Oct-1 plasmid. Luciferase activity was assessed by Dual-Luciferase® Reporter Assay System (Promega, Madison, WI, USA).

### Cell fractionation

Cytoplasmic and nuclear fractions were separated by using NE-PER Nuclear and Cytoplasmic Extraction Reagents Kit (Thermo, CA, USA).

### Gene expression analysis by PCR array

Capital Bio Mouse Genome Oligo Array from Capital Corporation was used for the examination of the expression pattern of genes involved in atherosclerosis. By using different arrays, the fold-change for aortic gene of *Apoe*^*−/−*^ *Ckip-1*^*−/−*^ mice with respect to *Apoe*^*−/−*^ mice on a 12-week Western diet was calculated. Data are expressed as mean ± s.e.m. of triplicate simples. Microarray data that support the findings of this study have been deposited in the Gene Expression Omnibus (GEO) under the accession code GSE109698.

### RNA-sequencing and gene expression analysis

The RNA-seq library was prepared for sequencing using standard Illumina protocols. Total RNA samples from *Ckip-1*^*−/−*^ BMDMS and WT BMDMs were isolated using TRIzol reagent (Invitrogen) and treated with RNase-free DNase I (New England Biolabs, MA, USA), to remove any contaminating genomic DNA. Library construction and sequencing were performed by Novogene (Beijing). For the data analysis, basecalls are performed using CASAVA. Clean reads were aligned to the genome using STAR (v2.5.1b) and HTSeq v0.6.0 was used to count the reads numbers mapped to each gene. Differential expression was determined using the edgeR package and the significance of the differential expression of genes was defined by the bioinformatics service according to the combination of the absolute value of log2-foldchange ≥ 1 and *P* value ≤ 0.05. GO, and pathway annotation and enrichment analyses were based on the Gene Ontology Database (http://www.geneontology.org/), and KEGG pathway database (http://www.genome.jp/kegg/), respectively. The software Cluster and Java Treeview were used for hierarchical cluster analysis of gene expression patterns. The original sequence data have been submitted to the database of the NCBI Sequence Read Archive (http://trace.ncbi.nlm.nih.gov/traces/sra) under the accession number PRJNA478820.

### Statistical analysis

Data are presented as mean ± s.e.m. The statistical significance of differences was evaluated with the Student’s *t* test or one-way analysis of variance (ANOVA). All statistical analyses were performed with GraphPad Prism 6 and SPSS 22.0 software. Significance was accepted at the level of *P* < 0.05. The resource data are shown in Supplementary Data 2.

### Reporting Summary

Further information on experimental design is available in the Nature Research Reporting Summary linked to this Article.

## Supplementary Information

Supplementary Information

Peer Review File

Description of Additional Supplementary Files

Supplementary Data 1

Supplementary Data 2

Reporting Summary

## Data Availability

The RNA-seq data in WT and *Ckip-1*^*−/−*^ BMDMs have been submitted to the database of the NCBI Sequence Read Archive under the accession number PRJNA478820. Microarray data that support the findings of this study have been deposited in the Gene Expression Omnibus under the accession code GSE109698. A Reporting Summary for this Article is available as a Supplementary Information file. The authors declare that all the relevant data supporting the findings of this study are available within the Article and its Supplementary Information files, or from the corresponding author on reasonable request.
