# Author Correction: CKIP-1 limits foam cell formation and inhibits atherosclerosis by promoting degradation of Oct-1 by REGγ

**DOI:** 10.1038/s41467-021-25986-6

**Published:** 2021-09-22

**Authors:** Jiao Fan, Lifeng Liu, Qingyan Liu, Yu Cui, Binwei Yao, Minghua Zhang, Yabing Gao, Yesheng Fu, Hongmiao Dai, Jingkun Pan, Ya Qiu, Cui Hua Liu, Fuchu He, Yu Wang, Lingqiang Zhang

**Affiliations:** 1 State Key Laboratory of Proteomics, National Center of Protein Sciences (Beijing), Beijing Institute of Lifeomics, Beijing, 100850 China; 2 Institute of Geriatrics, National Clinical Research Center of Geriatrics Disease, Chinese PLA General Hospital, Beijing, 100853 China; 3 Department of Cardiology, Chinese PLA General Hospital, Beijing, 100853 China; 4 Center of Therapeutic Research for Liver Cancer, 302 Military Hospital of China, Beijing, 100039 China; 5 Department of Experimental Pathology, Beijing Institute of Radiation Medicine, Beijing, 100850 China; 6 Clinical Pharmacy Laboratory, Chinese PLA General Hospital, Beijing, 100853 China; 7 CAS Key Laboratory of Pathogenic Microbiology and Immunology, Institute of Microbiology, Chinese Academy of Sciences, Beijing, 100101 China

**Keywords:** Foam cells, Atherosclerosis

Correction to: *Nature Communications* 10.1038/s41467-018-07895-3, published online 25 January 2019.

The original version of the Supplementary information associated with this Article contained an error in Supplementary Fig. 1a, in which the blots for β-actin in the left panel and the blots for CKIP-1 and β-actin in the right panel were inadvertently replaced with incorrect blots. The HTML has been updated to include a corrected version of the Supplementary information.

## Supplementary information

Updated Supplementary Information

