# Supplementary material for: CKIP-1 limits foam cell formation and inhibits atherosclerosis by promoting degradation of Oct-1 by REGγ
Source: Nat Commun. 2019 Jan 25;10:425. doi: 10.1038/s41467-018-07895-3 (PMC6347643; doi:10.1038/s41467-018-07895-3)
Supplement: Supplementary file 1 — Supplementary Information [file 41467_2018_7895_MOESM1_ESM.pdf]

CKIP-1 limits foam cell formation and inhibits atherosclerosis by promoting degradation of Oct-1 by REGγ

Jiao Fan et al.

Supplementary Figures

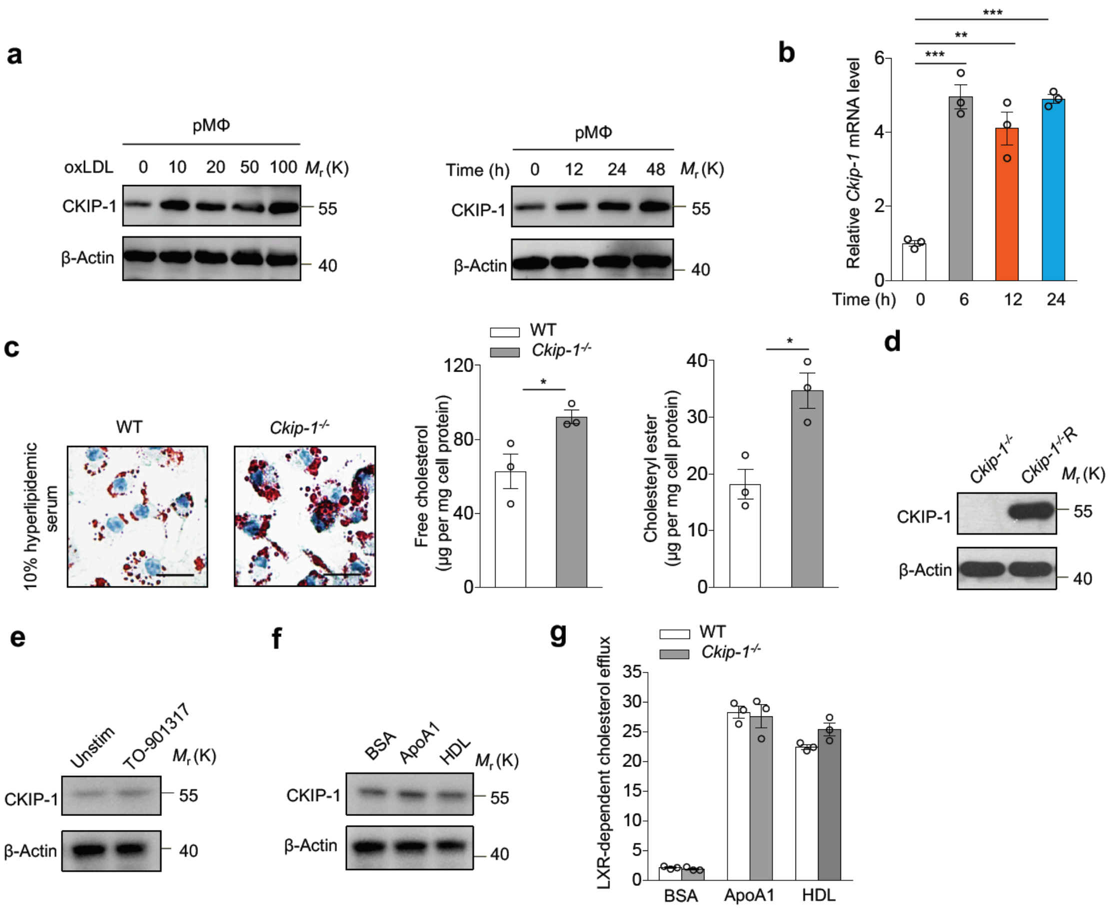

Supplementary Figure 1 CKIP-1 is critical for macrophage cholesterol accumulation and foam cell formation. (**a**) The expression of CKIP-1 was assessed by western blot in peritoneal macrophages (pMΦ) incubated with oxLDL (50 μg per ml) for indicated time and in pMΦ exposed to different doses of oxLDL for 24 hr. (**b**) mRNA levels of CKIP-1 in pMΦ after incubation with oxLDL (50 μg per ml) for indicated time. (**c**) Increased foam cell formation and accumulation of unesterified cholesterol and cholesteryl ester in *Ckip-1*$^{-/-}$ BMDMs after incubation in RPMI-1640 medium containing 10% serum from hyperlipidemic *Apoe*$^{-/-}$ mice for 24 hr. Scale bar, 25 μm. (**d**) Restoration of CKIP-1 to *Ckip-1*$^{-/-}$ BMDMs was confirmed by western blot. (**e**) CKIP-1 expression was assessed by western blot in BMDMs incubated with or without 1μM TO-901317 for 24 hr. (**f**) CKIP-1 expression was assessed by western blot when cholesterol efflux to BSA, ApoA1 and HDL from BMDMs was induced. (**g**) LXR-dependent cholesterol efflux to BSA, ApoA1 and HDL in macrophages from each group was detected. Data represent mean ± s.e.m. of n = 3 biologically independent experiments (**b**,**c**,**g**). P values were calculated by two-way ANOVA (**b**) and two-tailed Student's t-test (**c**,**g**). *P< 0.05, **P< 0.01, ***P< 0.001. The precise P value and statistics source data are in Supplementary Data 2. Unprocessed original scans of blots are shown in Supplementary Fig. 6.

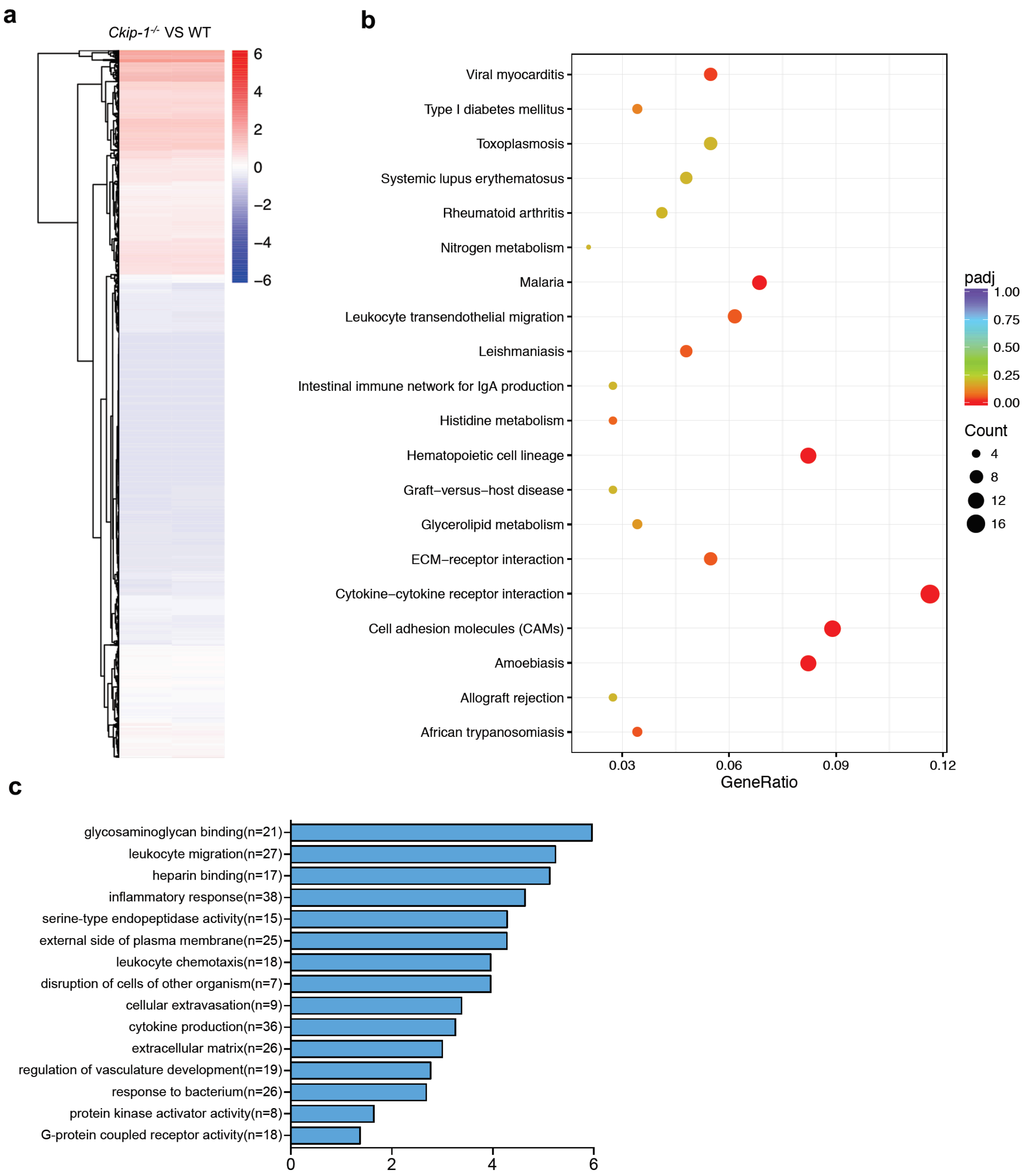

Supplementary Figure 2 WT and *Ckip-1*$^{-/-}$ macrophages were analyzed RNA sequencing. (**a**) Differentially expressed genes are shown as a heatmap. (**b**) KEGG analysis of the DEGs. (**c**) Gene Ontology (GO) enrichment analysis for biological process, cell component and molecular function of up-regulated proteins. The -$\log_{10}$ (*P* value) of enrichment is shown on x axis; the number of associated proteins for each term is shown on y axis.

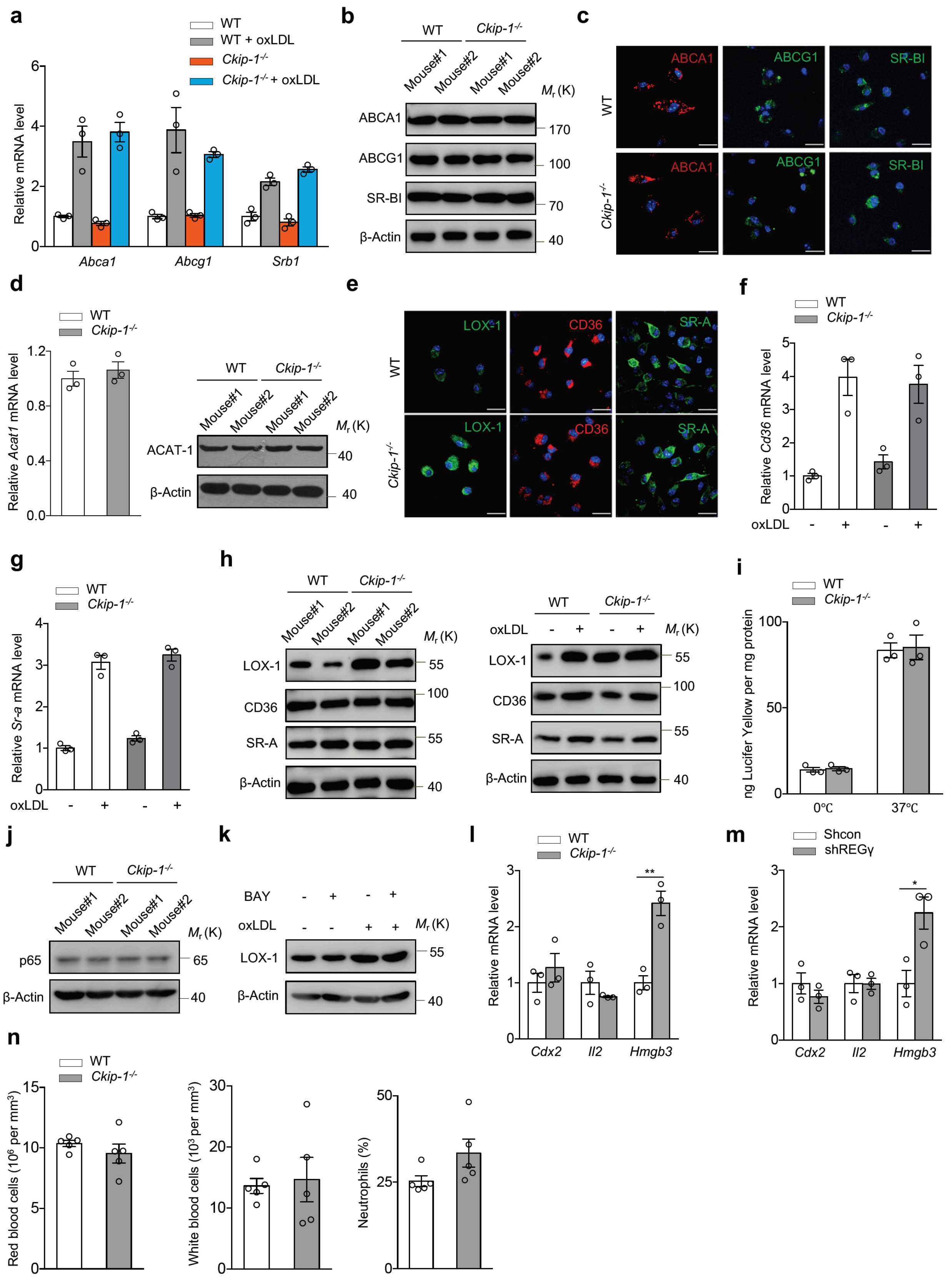

Supplementary Figure 3 *Ckip-1* deficiency promotes expression of scavenger receptor LOX-1. (**a-c**) Expression analysis of ABCA1, ABCG1 and SR-BI in WT and *Ckip-1*$^{-/-}$ macrophages at mRNA levels by RT-PCR (**a**), protein

levels by western blot (**b**) and immunofluorescence staining (**c**). Scale bar, 20 μm. (**d**) Analysis of mRNA levels and protein levels of ACAT-1 in WT and *Ckip-1*$^{-/-}$ macrophages. (**e**) Immunofluorescence staining of LOX-1, CD36 and SR-A in WT and *Ckip-1*$^{-/-}$ macrophage. (**f-g**) mRNA levels of CD36 and SR-A in BMDMs after incubation with or without oxLDL (20 μg per ml) for 24 hr. (**h**) Expression of LOX-1, CD36 and SR-A in WT and *Ckip-1*$^{-/-}$ pMΦ after 24 hr incubation with or without 20 μg per ml oxLDL. (**i**) Measure of pinocytosis by quantitative analysis of the lucifer yellow fluid-phase dye uptake in WT and *Ckip-1*$^{-/-}$ macrophages. (**j**) Expression analysis of p65 in WT and *Ckip-1*$^{-/-}$ BMDMs. (**k**) *Ckip-1*$^{-/-}$ BMDMs were treated with or without NF-κB inhibitor BAY11-7082 (10 μM) for 1 hr and then stimulated with oxLDL (50 μg per ml) for 24 hr and protein levels of LOX-1 were assessed. (**l**) Analysis of mRNA levels of Cdx-2, IL-2 and HMGB3 in WT and *Ckip-1*$^{-/-}$ macrophages. (**m**) Knockdown of REGγ in macrophages and then analysis of mRNA levels of Cdx-2, IL-2 and HMGB3. (**n**) Complete blood cell analysis of peripheral blood for red blood cells, white blood cells and neutrophils was performed on WT and *Ckip-1*$^{-/-}$ mice. Mice were anesthetized and peripheral blood was collected in ethylenediaminetetraacetic acid (EDTA)-covered Microvette collection tubes for complete blood cell analysis on fully automatic hematology analyzer. n = 5. Data represent mean ± s.e.m. of n = 3 biologically independent experiments (**a**,**d**,**f**,**g**,**i**,**l**,**m**). P values were calculated by two-tailed Student's t-test (**a**,**d**,**f**,**g**,**i**,**l**,**m**,**n**). *P< 0.05, **P< 0.01, ***P< 0.001. The precise P value and statistics source data are in Supplementary Data 2. Unprocessed original scans of blots are shown in Supplementary Fig. 6.

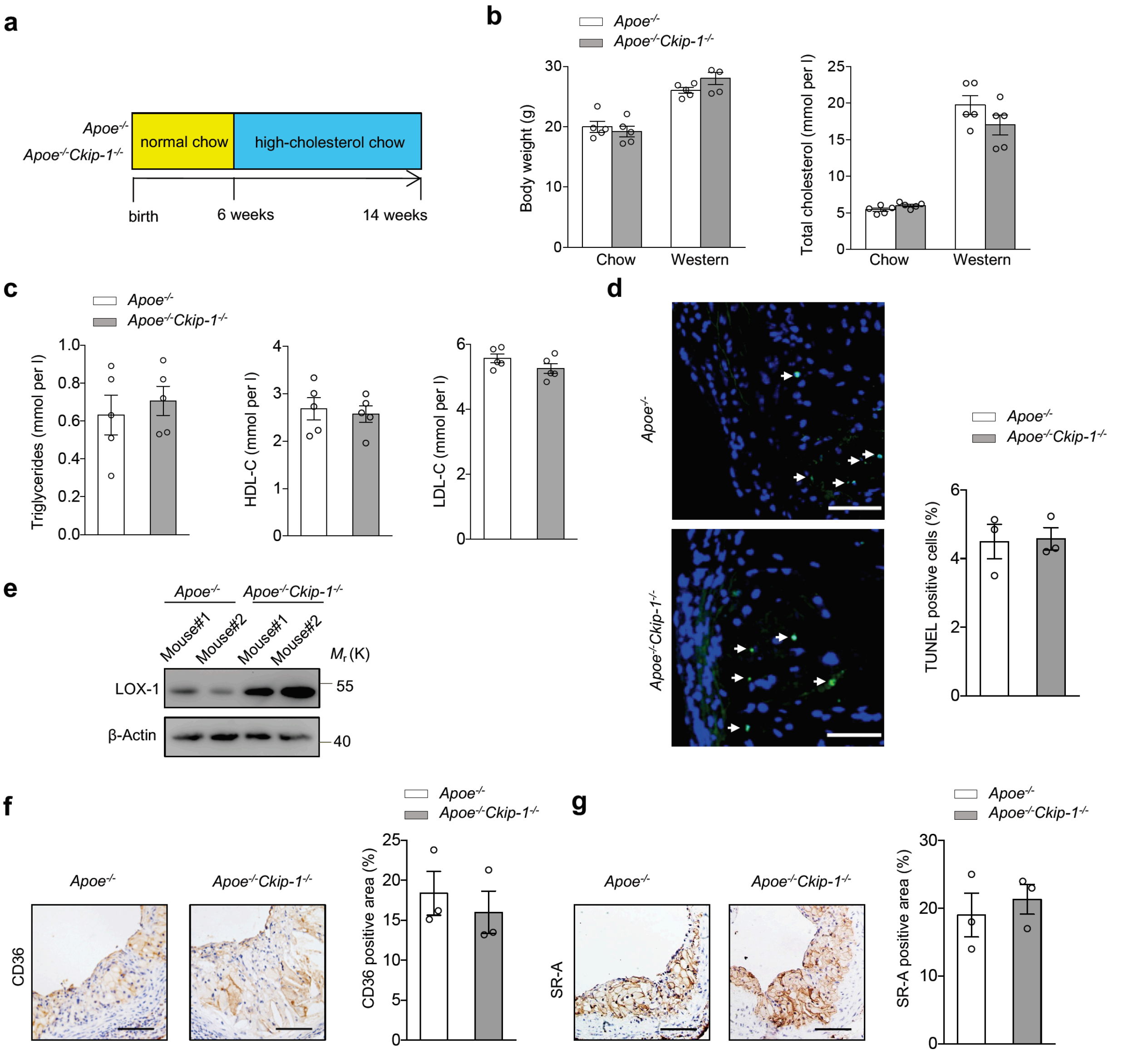

Supplementary Figure 4 Loss of *Ckip-1* promotes atherosclerosis in hyperlipidemic *Apoe*$^{-/-}$ mice. (**a**) Diet schemes. Eight weeks after birth, *Apoe*$^{-/-}$ and *Apoe*$^{-/-}$ *Ckip-1*$^{-/-}$ mice were fed a Western diet for 8 weeks. Sixteen-week-old animals were euthanized for tissue harvesting. (**b**) Body weight and total cholesterol levels from *Apoe*$^{-/-}$ and *Apoe*$^{-/-}$ *Ckip-1*$^{-/-}$ mice with or without Western diet. $n = 5$. (**c**) The levels of fasting triglycerides and lipoprotein profiles of *Apoe*$^{-/-}$ and *Apoe*$^{-/-}$ *Ckip-1*$^{-/-}$ mice after being fed a Western diet for 8 weeks. $n = 5$. Data represent mean ± s.e.m.. (**d**) TUNEL staining positive cells in lesions of *Apoe*$^{-/-}$ and *Apoe*$^{-/-}$ *Ckip-1*$^{-/-}$ mice on a Western diet for 9 weeks were detected by immunofluorescence. Scale bar, 50 µm. $n = 3$. (**e**) LOX-1 expression was detected by western blot in peritoneal macrophages isolated from *Apoe*$^{-/-}$ mice and *Apoe*$^{-/-}$ *Ckip-1*$^{-/-}$ mice on a 8-week Western diet. (**f**) Immunohistochemical detections of CD36 in aortas. Scale bars, 100 µm. $n = 3$. (**g**) Immunohistochemical detections of SR-A in aortas. Scale bars, 100 µm. $n = 3$. Data represent mean ± s.e.m.. *P* values were calculated by two-tailed Student's *t*-test (**b**,**c**,**d**,**f**,**g**). *$P < 0.05$, **$P < 0.01$, ***$P < 0.001$. The precise *P* value and statistics source data are in Supplementary Data 2. Unprocessed original scans of blots are shown in Supplementary Fig. 6.

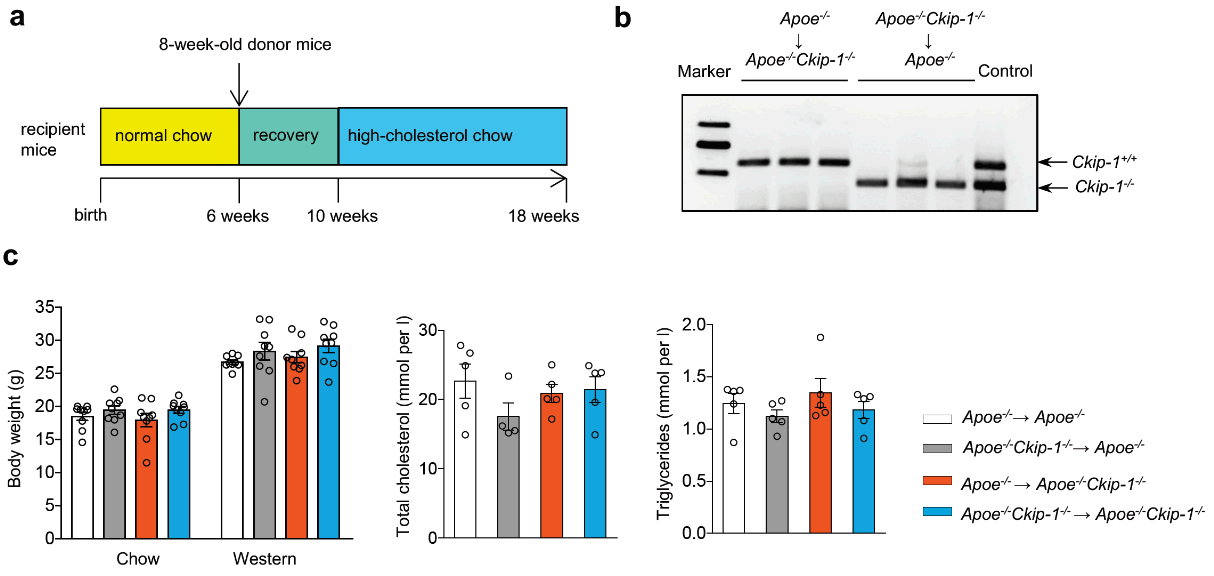

Supplementary Figure 5 Deletion of *Ckip-1* on hematopoietic cells is responsible for the increased atherosclerosis in *Apoe*$^{-/-}$ *Ckip-1*$^{-/}$ mice. (**a**) Diet schemes. Bone-marrow from 8-week-old donor mice was extracted, and 10^7 bone-marrow cells were injected intravenous into irradiated recipient mice. Transplanted mice were allowed to recover for 4 weeks, and were then fed a Western diet for 8 weeks prior to tissue harvesting. (**b**) Genotyping results with blood DNA sample from *Apoe*$^{-/-}$→ *Apoe*$^{-/-}$ *Ckip-1*$^{-/-}$ and *Apoe*$^{-/-}$ *Ckip-1*$^{-/-}$→ *Apoe*$^{-/-}$ mice. A tail DNA sample from a *Ckip-1*$^{+/-}$ mouse was presented as control. The upper band: WT allele; the lower band: *Ckip-1*$^{-/-}$ allele. (**c**) Body weight of mice with the indicated group before and after being fed an 8-week Western diet, $n = 9$. Total cholesterol levels and fasting triglycerides levels of each group after being fed a Western diet for 8 weeks, $n = 5$. Data represent mean ± s.e.m.. *P* values were calculated by two-tailed Student's *t*-test (**b**,**c**). *$P< 0.05$, **$P< 0.01$, ***$P< 0.001$. The precise *P* value and statistics source data are in Supplementary Data 2.

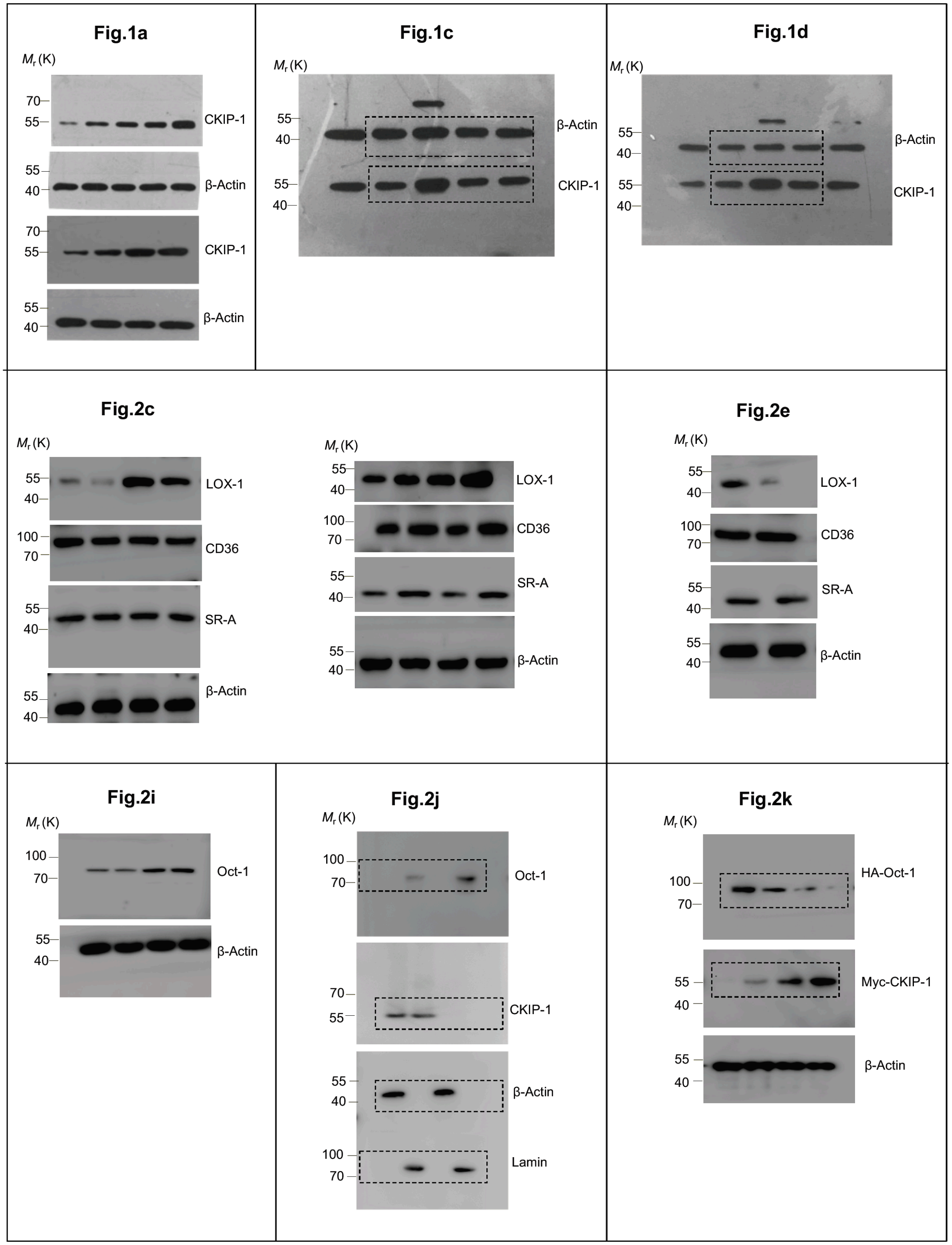

Supplementary Figure 6 Uncropped scans of membranes and gels used in the main and supplementary figures.

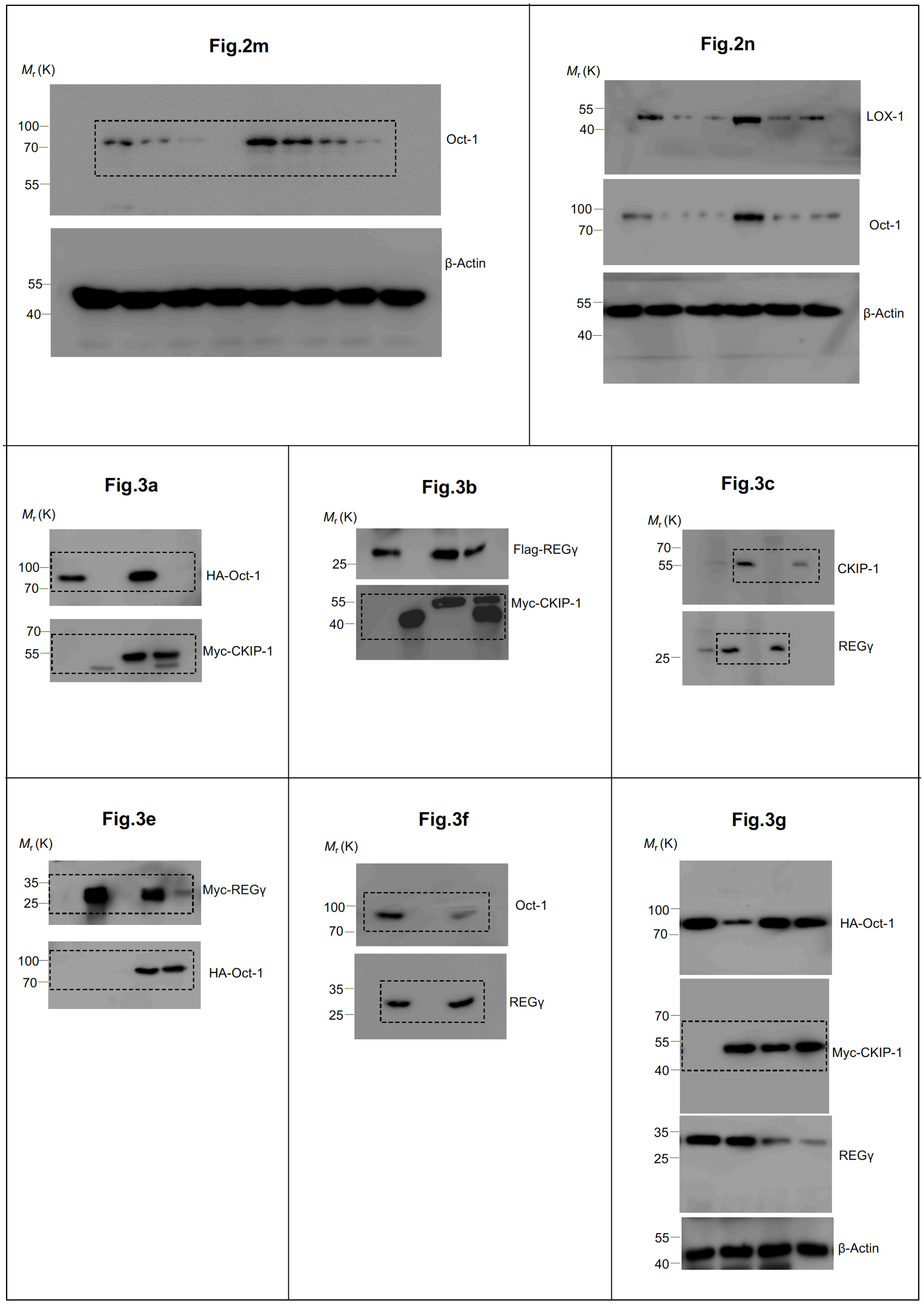

Supplementary Figure 6 Continued

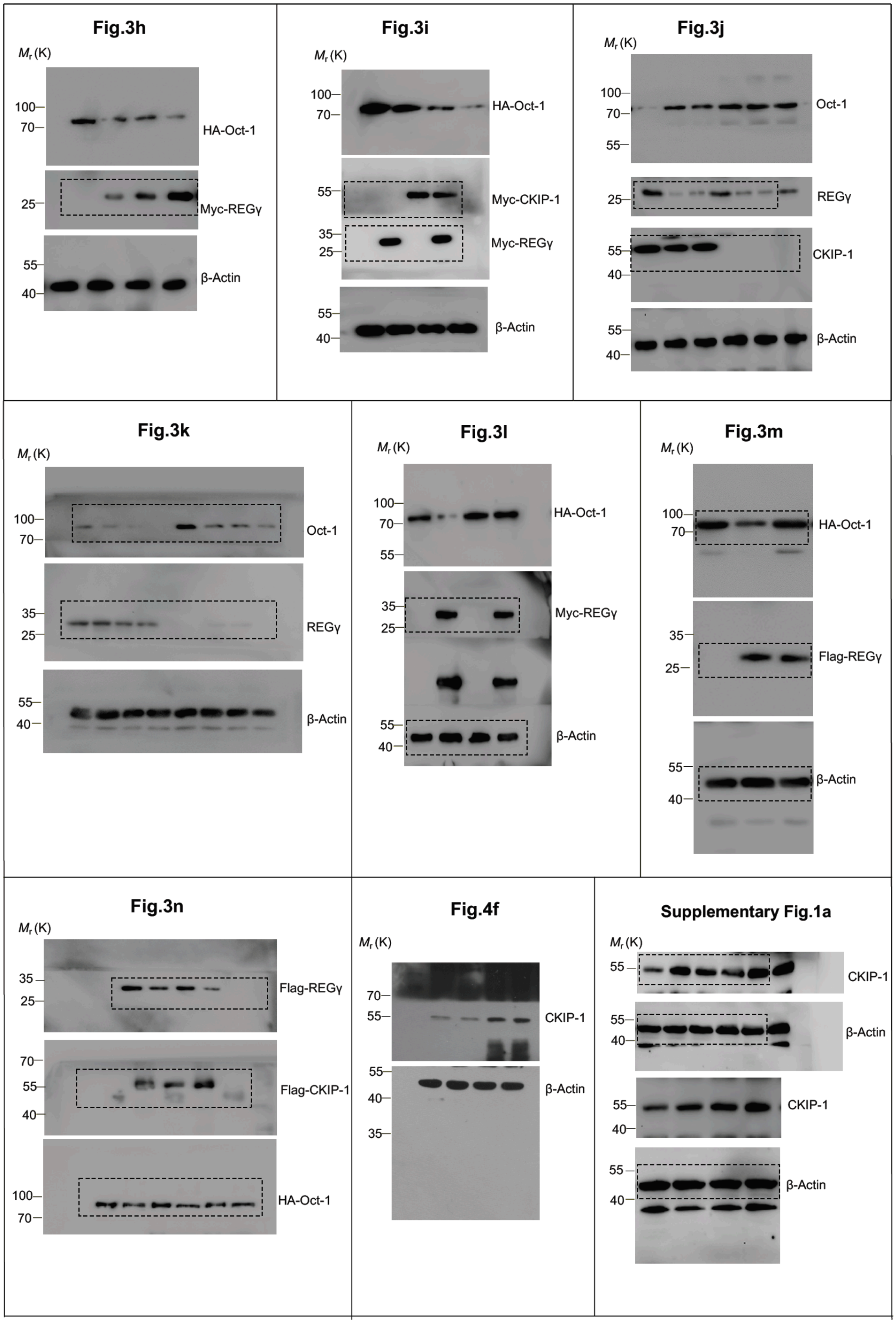

Supplementary Figure 6 Continued

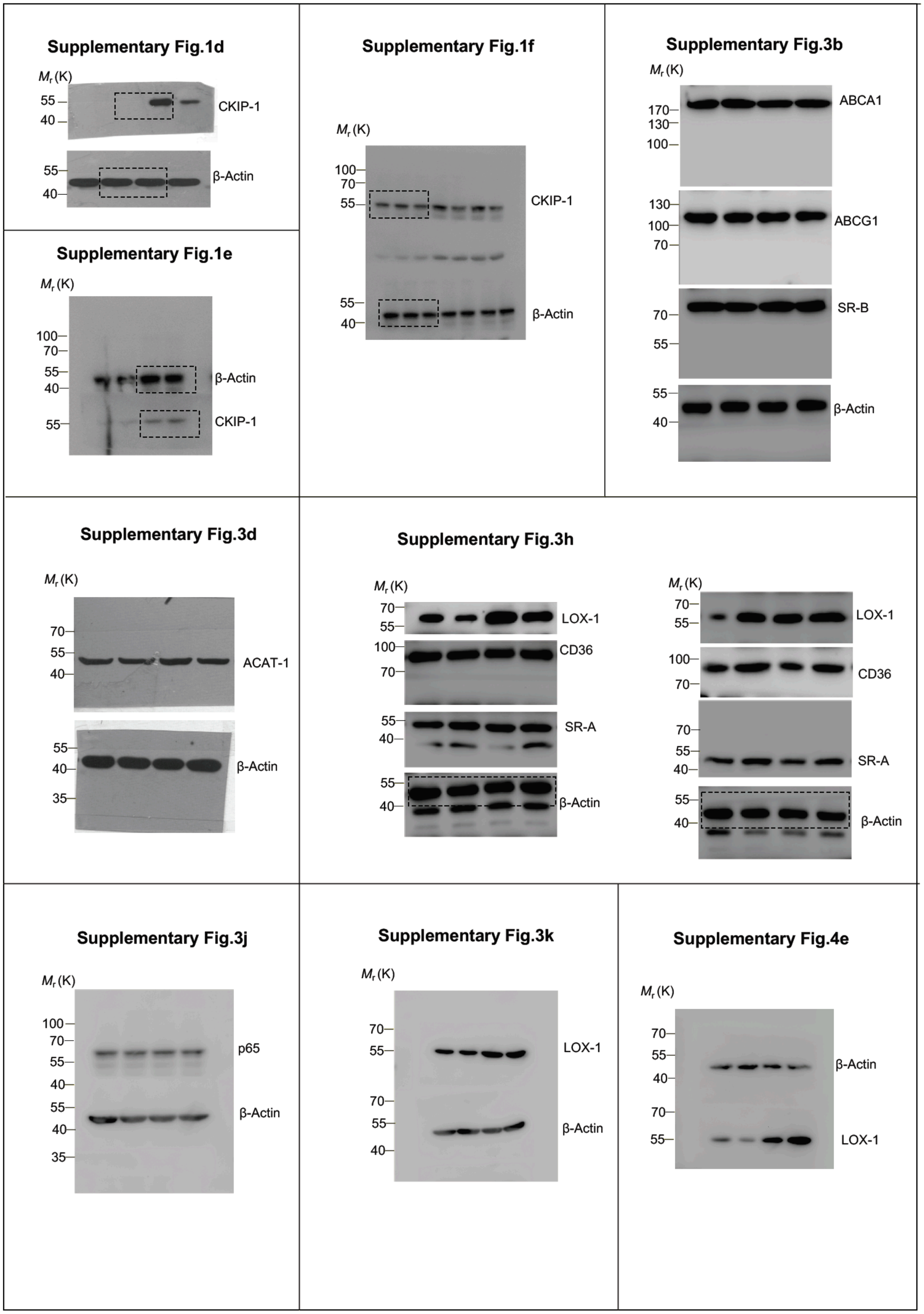

Supplementary Figure 6 Continued

Supplementary Tables

Supplementary Table 1: Yeast-two-hybrid screening results using CKIP-1 as bait

Gene	Accession	Description	CDS	Hits
BHLHB2	NM_003670	Homo sapiens basic helix-loop-helix domain containing, class B2	(197-1435)	1
C16orf35	NM_001077350	Homo sapiens chromosome 16 open reading frame 35	(100-1809)	1
DAZAP2	NM_014764	Homo sapiens DAZ associated protein 2	(67-573)	1
EFEMP1	NM_001039348	Homo sapiens EGF-containing fibulin-like extracellular matrix protein 1	(136-1617)	1
FBLN1	NM_001996	Homo sapiens fibulin 1, transcript variant C	(148-2199)	1
FRS3	NM_006653	Homo sapiens fibroblast growth factor receptor substrate 3	(245-1723)	1
GFAP	NM_002055	Homo sapiens glial fibrillary acidic protein	(15-1313)	1
HNRPK	NM_002140	Homo sapiens heterogeneous nuclear ribonucleoprotein, transcript variant 1	(210-1604)	1
NEFL	NM_006158	Homo sapiens neurofilament, light polypeptide 68 kDa	(98-1729)	3
NELL2	NM_006159	Homo sapiens NEL-like 2	(97-2547)	1
PET112L	NM_004564	Homo sapiens PET112-like	(13-1686)	1
PSMC5	NM_002805	Homo sapiens proteasome 26S subunit, ATPase, 5	(42-1262)	1
PSME3	NM_005789	Homo sapiens proteasome activator subunit3 (PA28 gamma; Ki)	(227-991)	1
SF3B4	NM_005850	Homo sapiens splicing factor 3b, subunit 4	(52-1326)	1
TICAM1	NM_182919	Homo sapiens toll-like receptor adaptor molecule 1	(63-2201)	1

Supplementary Table 2: Sequences for RT-PCR primers used in this study

Gene	Forward (5'->3')	Reverse (5'->3')
CKIP-1	AATTCTGCGGGAAAGGGATTT	AACACCTCCTGACTGTTTTCTC
LOX-1	TCTTCCATGGGCCCTTTAGCTG	TTCCGATGCAATCCAATCCAGA
CD36	TCCTATTGGCCAAGCTATTGCG	CACGGGGATTCCTTTAAGGTCG
SR-A	AAAGAAGAACAAGCGCACGTGG	GAGCACCAGGTGGACCAGTTTG
ABCA1	CGTTTCCGGGAAGTGTCCTA	GCTAGAGATGACAAGGAGGATGGA
ABCG1	GGGAAGTTGATAAAGGATGT	GATTCGGGCTATGTATGG
SR-B1	CAGTAGTTCTGCCGTTGCTG	TGAATGGCCTCCTTATCCTG
CD68	TGTCTGATCTTGCTAGGACCG	GAGAGTAACGGCCTTTTTGTGA
Oct-1	CCTGCAACCAGCACAGTTTA	CTACGATTCAAGCCCTCAGC
ACAT-1	CAGAAATGGTCACATTACCATGAAACA	TGCTTGATTTCTCCTTGGCTGCATTTAGT
HMGB3	AGAGGTGGAAGACCATGTC	TCTCCCGATCATATCGGAC
Cdx-2	AGTGAGCTGGCTGCCACACT	GCTGCTGCTGCTTCTTCTTGA
IL-2	TCCAGAACATGCCGCAGAG	CCTGAGCAGGATGGAGAATTACA
CCL-2	TTAAAAACCTGGATCGGAACCAA	GCATTAGCTTCAGATTTACGGGT
IL-1β	GCCCATCCTCTGTGACTCA	AGGCCACAGGTATTTTGTCG
IL-6	TAGTCCTTCCTACCCCAATTTCC	TTGGTCCTTAGCCACTCCTTC
VCAM-1	TGATTGGGAGAGACAAAGCA	AACAACCGAATCCCCAACTT
ICAM-1	TGGCCTGGGGGATGCACACT	GGCTGTAGGTGGGTCCGGGT
β-actin	GAGCGTGGCTACAGCTTCAC	GGCATAGAGGTCTTTACGGATG
