# Supplementary material for: CKIP-1 limits foam cell formation and inhibits atherosclerosis by promoting degradation of Oct-1 by REGγ
Source: Nat Commun. 2019 Jan 25;10:425. doi: 10.1038/s41467-018-07895-3 (PMC6347643; doi:10.1038/s41467-018-07895-3)
Supplement: Supplementary file 3 — Description of Additional Supplementary Files [file 41467_2018_7895_MOESM3_ESM.pdf]

Description of Additional Supplementary Files

File Name: Supplementary Data 1

Description: RNA-seq analysis: DEGs of Ckip-1–/– and WT BMDMs

File Name: Supplementary Data 2

Description: Statistics source data
