# Supplementary material for: CKIP-1 limits foam cell formation and inhibits atherosclerosis by promoting degradation of Oct-1 by REGγ
Source: Nat Commun. 2019 Jan 25;10:425. doi: 10.1038/s41467-018-07895-3 (PMC6347643; doi:10.1038/s41467-018-07895-3)
Supplement: Supplementary file 6 — Reporting Summary [file 41467_2018_7895_MOESM6_ESM.pdf]

nature research

Corresponding author(s): Lingqiang Zhang

Reporting Summary

Nature Research wishes to improve the reproducibility of the work that we publish. This form provides structure for consistency and transparency in reporting. For further information on Nature Research policies, see Authors & Referees and the Editorial Policy Checklist.

Statistical parameters

When statistical analyses are reported, confirm that the following items are present in the relevant location (e.g. figure legend, table legend, main text, or Methods section).

n/a	Confirmed	
☐	☒	The exact sample size (*n*) for each experimental group/condition, given as a discrete number and unit of measurement
☐	☒	An indication of whether measurements were taken from distinct samples or whether the same sample was measured repeatedly
☐	☒	The statistical test(s) used AND whether they are one- or two-sided<br>*Only common tests should be described solely by name; describe more complex techniques in the Methods section.*
☐	☒	A description of all covariates tested
☒	☐	A description of any assumptions or corrections, such as tests of normality and adjustment for multiple comparisons
☐	☒	A full description of the statistics including central tendency (e.g. means) or other basic estimates (e.g. regression coefficient) AND variation (e.g. standard deviation) or associated estimates of uncertainty (e.g. confidence intervals)
☐	☒	For null hypothesis testing, the test statistic (e.g. *F*, *t*, *r*) with confidence intervals, effect sizes, degrees of freedom and *P* value noted<br>*Give P values as exact values whenever suitable.*
☒	☐	For Bayesian analysis, information on the choice of priors and Markov chain Monte Carlo settings
☒	☐	For hierarchical and complex designs, identification of the appropriate level for tests and full reporting of outcomes
☒	☐	Estimates of effect sizes (e.g. Cohen's *d*, Pearson's *r*), indicating how they were calculated
☐	☒	Clearly defined error bars<br>*State explicitly what error bars represent (e.g. SD, SE, CI)*

Our web collection on statistics for biologists may be useful.

Software and code

Policy information about availability of computer code

Data collection	No software was used.
Data analysis	"Graphad Prism 6" and "SPSS 22.0" software were used to analyze the data in this study.

For manuscripts utilizing custom algorithms or software that are central to the research but not yet described in published literature, software must be made available to editors/reviewers upon request. We strongly encourage code deposition in a community repository (e.g. GitHub). See the Nature Research guidelines for submitting code & software for further information.

Data

Policy information about availability of data

All manuscripts must include a data availability statement. This statement should provide the following information, where applicable:

- Accession codes, unique identifiers, or web links for publicly available datasets
- A list of figures that have associated raw data
- A description of any restrictions on data availability

• The datasets generated during and/or analysed during the current study are available from the corresponding author on reasonable request.

Field-specific reporting

Please select the best fit for your research. If you are not sure, read the appropriate sections before making your selection.

☒ Life sciences ☐ Behavioural & social sciences ☐ Ecological, evolutionary & environmental sciences

For a reference copy of the document with all sections, see nature.com/authors/policies/ReportingSummary-flat.pdf

Life sciences study design

All studies must disclose on these points even when the disclosure is negative.

Sample size	Sample size was based on empirical data from pilot experiments.
Data exclusions	No data were excluded from the analysis.
Replication	Experimental findings were replicated successfully and data are presented as mean ± SEM.
Randomization	Atherosclerosis was induced by feeding gender-matched 8-week-old Apoe−/− mice and Apoe−/− Ckip-1−/−mice with a Western diet from Harlan Teklad (TD88137) for indicated times. No additional randomization or blinding was used to allocation during experiments and outcome assessment.
Blinding	Atherosclerosis was induced by feeding gender-matched 8-week-old Apoe−/− mice and Apoe−/− Ckip-1−/−mice with a Western diet from Harlan Teklad (TD88137) for indicated times. No additional randomization or blinding was used to allocation during experiments and outcome assessment.

Reporting for specific materials, systems and methods

Materials & experimental systems

n/a	Involved in the study
☒	☐ Unique biological materials
☐	☒ Antibodies
☐	☒ Eukaryotic cell lines
☒	☐ Palaeontology
☐	☒ Animals and other organisms
☒	☐ Human research participants

Methods

n/a	Involved in the study
☒	☐ ChIP-seq
☒	☐ Flow cytometry
☒	☐ MRI-based neuroimaging

Antibodies

Antibodies used	All antibodies were purchased as follows: Anti-CKIP-1 (sc-50225; for immunohistochemical analysis (IHC), 1:100 dilution; for immunofluorescent analysis (IF), 1:1000 dilution; for western blot analysis (WB), 1:500 dilution), anti-Oct-1 (sc-8024; for WB, 1:500 dilution; for immunoprecipitation analysis (IP), 1:50 dilution), anti-ABCG1 (sc-11150; for IF, 1:100 dilution; for WB, 1:200 dilution), anti-Lamin (sc-518013; for WB, 1:200 dilution), anti-SR-B (sc-32342; for IF, 1:100 dilution; for WB, 1:200 dilution) and anti-actin (sc-1616; for WB, 1:1000 dilution) antibodies were purchased from Santa Cruz. Anti-REGγ (ab157157; for WB, 1:500 dilution; for IP, 1:50 dilution; for IF, 1:100 dilution), anti-CD68 (ab125212; for IHC, 1:200 dilution; for IF, 1:200 dilution), anti-CD3 (ab16669; for IHC, 1:100 dilution), anti-SMA (ab9465; for IHC, 1:200 dilution), anti-ABCA1 (ab18180; for IF, 1:200 dilution; for WB, 1:200 dilution), and anti-ACAT-1 (ab168342; for WB, 1:500 dilution) antibodies were purchased from Abcam. Anti-LOX-1 (AF1564; for IHC, 1:200 dilution; for IF, 1:200 dilution; for WB, 1:1000 dilution), anti-CD36 (AF2519; for IHC, 1:200 dilution; for IF, 1:200 dilution; for WB, 1:1000 dilution), anti-SR-A (AF1797; for IHC, 1:200 dilution; for IF, 1:200 dilution; for WB, 1:1000 dilution), anti-MMP-9 (AF909; for IHC, 1:200 dilution), and anti-VCAM-1 (AF2519; for IF, 1:200 dilution) antibodies were purchased from R&D. Anti-HA (M180-3; for WB, 1:1000 dilution) and anti-Myc (M047-3; for WB, 1:1000 dilution) antibodies were purchased from MBL. Anti-Flag (F7425; for WB, 1:1000 dilution) antibody was purchased from Sigma. Anti-NF-κB (8242; for WB, 1:1000 dilution) antibody was purchased from Cell Signaling Technology.
Validation	Anti-CKIP-1: Goat, for IHC, IF, WB; anti-Oct-1: Mouse, for WB, IP; anti-ABCG1: Goat, for IF, WB; anti-Lamin: Mouse, for WB; anti-SR-B: Goat, for IF, WB; anti-actin: Mouse, for WB; Anti-REGγ: Rabbit, for WB, IP, IF; anti-CD68: Rabbit, for IHC, IF; anti-CD3:Rabbit, for IHC; anti-SMA: Rabbit, for IHC; anti-ABCA1: Mouse, for IF, WB; anti-ACAT-1: Rabbit, for WB; anti-LOX-1: Goat, for IHC, WB, IF; anti-CD36: Goat, for IHC, WB, IF; anti-SR-A: Goat, for IHC, WB, IF; anti-MMP-9: Goat, for IHC; anti-VCAM-1: Goat, for IF; anti-HA: Mouse, for WB; anti-Myc: Mouse,for WB; Anti-Flag: Mouse, for WB; Anti-NF-κB: Rabbit, for WB.

Eukaryotic cell lines

Policy information about cell lines

Cell line source(s)	HEK293T (ATCC CRL-3216) and HeLa (ATCC CCL-2) were obtained from the American Type Culture Collection (ATCC).
Authentication	We purchased the cell lines from the American Type Culture Collection (ATCC).
Mycoplasma contamination	The cell lines were tested for mycoplasma contamination using MycoAlert Mycoplasma Detection Kit (Lonza, # LT07-418).
Commonly misidentified lines (See ICLAC register)	No commonly misidentified cell line is used.

Animals and other organisms

Policy information about studies involving animals; ARRIVE guidelines recommended for reporting animal research

Laboratory animals	Ckip-1−/− mice (C57BL/6 background) were intercrossed with Apoe−/− mice36 (C57BL/6 background) to generate Apoe−/−mice and Apoe−/−Ckip-1−/− littermate controls. Atherosclerosis was induced by feeding gender-matched 8-week-old Apoe−/− mice and Apoe−/− Ckip-1−/− mice with a Western diet from Harlan Teklad (TD88137) for indicated times.
Wild animals	The study did not involve wild animals.
Field-collected samples	The study did not involve samples collected from field.
