# Supplementary material for: CKIP-1 limits foam cell formation and inhibits atherosclerosis by promoting degradation of Oct-1 by REGγ
Source: Nat Commun. 2019 Jan 25;10:425. doi: 10.1038/s41467-018-07895-3 (PMC6347643; doi:10.1038/s41467-018-07895-3)
Supplement: Supplementary file 2 — Peer Review File [file 41467_2018_7895_MOESM2_ESM.docx]

This manuscript has been previously reviewed at another journal that is not operating a transparent peer review scheme. This document only contains reviewer comments and rebuttal letters for versions considered at *Nature Communications*.

REVIEWERS' COMMENTS:

Reviewer #1 (Remarks to the Author):

I was invited to review this revised manuscript after it had already gone through at least one round of review. The authors have provided additional solid data (RNA-seq, NF-κB, ect) to support the

concept that CKIP-1 plays an important role in atherosclerotic development through accelerating the Oct-

have already carefully answered my questions by point-by point.

My minor question is regarding the presentation of Oct-1 expression in Figure 2I and 3K. When they analyzed the Oct-1 protein levels at the indicated time points after CHX treatment, that meant they observed the degradation of Oct-1. Since the Oct-1 expression levels were higher in CKIP-1 KO and

axis for each group demonstrated a RELATIVE Oct-1 expression normalized to 0 time point OR Oct-1 degradation (%). Otherwise the readers will be confused.

Reviewer #2 (Remarks to the Author):

The authors have responded extensively to the reviewer comments from the review at NCB. The manuscript is greatly improved. I have no further comments.

RESPONSE TO REVIEWERS' COMMENTS:

Reviewer #1 (Remarks to the Author):

# I was invited to review this revised manuscript after it had already gone through at least one round of review. The authors have provided additional solid data (RNA-seq, NF-κB, ect) to support the concept that CKIP-1 plays an important role in atherosclerotic development through accelerating the Oct-1 degradation by interacting REGγ. The current version has been largely improved, in which they have already carefully answered my questions by point-by point. My minor question is regarding the presentation of Oct-1 expression in Figure 2I and 3K. When they analyzed the Oct-1 protein levels at the indicated time points after CHX treatment, that meant they observed the degradation of Oct-1. Since the Oct-1 expression levels were higher in CKIP-1 KO and REGγ knockdown cells compared to control groups (the baselines are different), the set with 1.0 in Y axis for each group demonstrated a RELATIVE Oct-1 expression normalized to 0 time point OR Oct-1 degradation (%). Otherwise the readers will be confused.

Response: Thank you very much for your comments. According to your advices, we have changed the label of Y axis from “Oct-1 relative expression” into “Oct-1 degradation (%) ” .

Reviewer #2 (Remarks to the Author):

# The authors have responded extensively to the reviewer comments from the review at NCB. The manuscript is greatly improved. I have no further comments.

Response: We sincerely thank the reviewer for the kind comments and the recommendation for publication.
